# Hybrid Fluorescent Mass-Tag Nanotrackers as Universal Reagents for Long-Term Live-Cell Barcoding

**DOI:** 10.1021/acs.analchem.2c00795

**Published:** 2022-07-22

**Authors:** Antonio Delgado-Gonzalez, Jose Antonio Laz-Ruiz, M. Victoria Cano-Cortes, Ying-Wen Huang, Veronica D. Gonzalez, Juan Jose Diaz-Mochon, Wendy J. Fantl, Rosario M. Sanchez-Martin

**Affiliations:** † GENYO, Centre for Genomics and Oncological Research, Pfizer/University of Granada/Andalusian Regional Gov-ernment, PTS Granada, Avda. Ilustración 114, 18016 Granada, Spain; ‡ Department of Medicinal & Organic Chemistry and Excellence Research Unit of “Chemistry applied to Biomedi-cine and the Environment”, Faculty of Pharmacy, University of Granada, Campus de Cartuja s/n, 18071 Granada, Spain; § Biosanitary Research Institute of Granada (ibs.GRANADA), University Hospitals of Granada-University of Grana-da, 18012 Granada, Spain; ∥ Department of Urology, Stanford University School of Medicine, Stanford, California 94305, United States; ⊥ Stanford Cancer Institute, Stanford University School of Medicine, Stanford, California 94305, United States; # Department of Obstetrics and Gynecology, Stanford University School of Medicine, Stanford, California 94304, United States

## Abstract

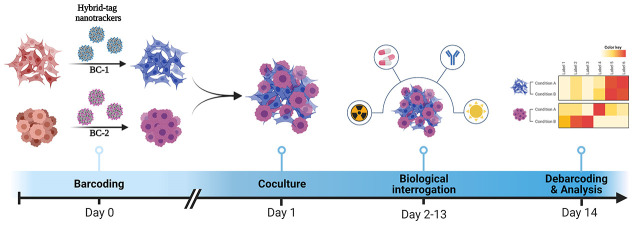

Barcoding and pooling cells for processing as a composite sample are critical to minimize technical variability in multiplex technologies. Fluorescent cell barcoding has been established as a standard method for multiplexing in flow cytometry analysis. In parallel, mass-tag barcoding is routinely used to label cells for mass cytometry. Barcode reagents currently used label intracellular proteins in fixed and permeabilized cells and, therefore, are not suitable for studies with live cells in long-term culture prior to analysis. In this study, we report the development of fluorescent palladium-based hybrid-tag nanotrackers to barcode live cells for flow and mass cytometry dual-modal readout. We describe the preparation, physicochemical characterization, efficiency of cell internalization, and durability of these nanotrackers in live cells cultured over time. In addition, we demonstrate their compatibility with standardized cytometry reagents and protocols. Finally, we validated these nanotrackers for drug response assays during a long-term coculture experiment with two barcoded cell lines. This method represents a new and widely applicable advance for fluorescent and mass-tag barcoding that is independent of protein expression levels and can be used to label cells before long-term drug studies.

## Introduction

Barcoding is essential for multiplex technologies to enhance sample consistency by minimizing technical issues arising from antibody staining, sample cross-contamination in the loading loop, and fluctuations in machine sensitivity. Additionally, a composite sample requires less time in the instrument together with reduced reagent consumption.^1^ Fluorescent cell barcoding has been established as a standard method to allow multiplexing in flow cytometry analysis.^2−4^ In parallel, mass cytometry (aka, cytometry by time-of-flight, CyTOF), a hybrid technology between flow cytometry and time-of-flight mass spectrometry, has enabled the implementation of alternative methods for efficient cell barcoding and multiplexing.^5−7^ Using antibodies tagged with stable metal isotopes (originally lanthanides), mass cytometry enables measurements of up to 60 parameters per single cell, with its greatest impact on revealing previously unrecognized levels of detail in heterogeneous cell populations.^8−13^ The mass cytometer readout spans 79–209 atomic mass units (amu) and measures many non-lanthanide metal isotopes in an ever-increasing number of reagents: antibody tags,^14,15^ metallointercalators,^16^ small-molecule probes,^1,17−22^ polymer-dots,^23^ inorganic nanoparticles,^12,24−27^ and polystyrene particles.^13,28,29^

The first generation of mass cytometry barcodes used lanthanides, with the limitation that the number of lanthanides available for tagging antibodies was reduced.^30^ The second generation of mass cytometry barcoding reagents used routinely and commercially available palladium isotopes whose atomic weights are well separated from those of the lanthanides. Using the six most abundant palladium isotopes, 20 unique barcodes were created using a doublet-free strategy (6-choose-3).^17^ However, these and other barcoding reagents created with different metals^23^ are intracellular labels and require fixation and permeabilization. This fact makes these reagents unsuitable for long-term culture assays. To overcome this issue, a large number of reagents were developed to barcode cell surface molecules such as CD45, the β-macroglobulin subunit of major histocompatibility complex (MHC) class 1, and the β-3 subunit of the sodium/potassium ATPase.^31−34^ On the other hand, cell-compatible small probes have been investigated such as osmium and ruthenium in the form of oxides^35^ or maleimide-functionalized tellurophene probe, TeMal,^36−38^ allowing cell barcoding not only in live cells but also in permeabilized cells. Notably, none of the barcoding reagents described above can be used in long-term drug response studies, whereby cells need to be prebarcoded just before a mass cytometry study begins.^39,40^ Some efforts have been focused on the development of dual reagents, but none has been reported so far for flow and mass cytometry dual-modal readout in longitudinal cell assays and multiplexed drug studies.^41,42^

The narrow size and highly uniform metal-loading capacity with minimal particle-to-particle variation of nanoparticles make them ideal analytes for mass cytometry. In this context, lanthanides have been conjugated into nanoparticles for cell labeling.^43^ On the other hand, lanthanide-infused polystyrene beads are routinely used in mass cytometry for instrument calibration and normalization.^13^ We previously reported the efficient conjugation and delivery of a variety of bioactive molecules such as drugs, proteins, nucleic acids, and other small molecules, using cross-linked polystyrene-based nanoparticles (NPs). These nanoparticles are characterized by their tunability, robustness with a defined loading capacity, and innocuousness. Polymeric nanoparticles have been used for imaging,^44,45^ biosensing,^46^ tracking of cellular proliferation,^47^*in cellulo* proteomics,^48^ and for selective delivery in a coculture approach based on the expression levels of cell surface receptors.^49^ In addition, we previously reported the preparation and validation of polystyrene beads containing palladium for intracellular catalysis, demonstrating their efficient transport into cells and innocuousness.^50,51^ Furthermore, a simplified synthesis protocol was optimized to generate dual polystyrene beads with a metal and a fluorophore to achieve efficient cellular analysis by mass cytometry and flow cytometry.^29^

Based on our expertise in generating versatile and biocompatible nanoparticles, we report the synthesis of fluorescent and palladium-based nanotrackers as hybrid-tag reagents for use in long-term cell culture as barcoding tools. We describe the methodology for the chemical synthesis and physicochemical characterization of these barcoding reagents and evaluate their compatibility with standard mass and flow cytometry reagents and their performance in biological systems. Then, we evaluated the suitability of nanotrackers for heterogeneous cell populations together with their dual application for mass and flow cytometry readout. Finally, a proof-of-principle study was carried out using two hybrid-tag nanotrackers to barcode two different cell populations in coculture by monitoring the biological response during long-term drug exposure. We found that our hybrid-tag nanotrackers are robust and versatile non-toxic live-cell barcoding reagents, compatible with protein characterization, and suitable tools for multiplexed drug studies and longitudinal cell assays.

## Experimental Section

### Materials

All solvents, chemicals, and reagents used in this work are detailed in the Supporting Information (SI).

### Preparation and Characterization of Hybrid-Tag Nanotrackers

Aminomethyl cross-linked polystyrene nanoparticles (NK-NPs (**1**)) were PEGylated; then, following Fmoc removal, cyanine conjugation step was carried out. Next, isotopically pure 10 mM Pd(NO_3_)_2_ (^106^Pd (99.3%) or ^110^Pd (99.4%)) in H_2_O was added to NPs. Pd(II) was reduced to Pd(0) by treatment with 10% hydrazine in methanol (MeOH) to achieve hybrid-tag nanotrackers. The physical–chemical characterization was achieved by measuring the particle mean size, size distribution, and ζ-potential of nanotrackers (NTs) by dynamic light scattering (DLS) and measured on a Zetasizer Nano ZS ZEN. The shape and morphology of the NTs were observed by ultrahigh-resolution transmission electron microscopy (HRTEM). Palladium presence was determined by HRTEM with an FEI microanalysis system for energy-dispersive X-ray (EDX), monitoring the profile of Pd 3d photoemission by obtaining its X-ray photoelectron spectra (XPS), and by mass cytometry. The fluorescence signal of nanotrackers was detected by flow cytometry and confocal microscopy.

### Live-Cell Barcoding Assays

Cells were incubated with a 2,500 NTs/cell ratio for 3 h. Then, cells were stained with cisplatin solution and fixed in 1.6% final concentration paraformaldehyde (PFA) in PBS. Cells were washed with cell staining medium (CSM) solution, permeabilized with ice-cold MeOH for 20 min, and stained with a cocktail of metal-labeled antibodies at RT for 1 h. Cells were labeled overnight with Intercalator-Ir (Fluidigm) (final concentration of 125 nM) in 1.6% PFA in PBS. Cells were washed with CyTOF water and resuspended in 0.1x normalization beads prior introduction into the mass cytometer. Flow cytometry standard (FCS) data sets were analyzed using Cytobank Community software. To assess the performance of these nanotrackers as live-cell barcodes of a heterogeneous population of blood cells, this protocol was slightly modified. Briefly, a preliminary step was carried out to lyse the erythrocytes with Quicklysis buffer (Cytognos), isolating the peripheral blood mononuclear cells (PBMCs). Then, following the barcoding step with either **BC-1** or **BC-2** (25,000 NTs/cell for 30 min), cells were incubated with CD45-FITC, CD3-APC-Cy7, and CD14-PerCP-Cy5.5 antibodies and sorted. Barcoded CD45+/CD3+ (T cells) and CD45+/CD14+ (monocytes) cells were isolated, then pooled and analyzed by mass cytometry, as described above. Monocytes and T cells without barcoding were used as control. The human blood samples from healthy donors were provided by the Biobank of the Andalusian Public Health System (agreement number S2100107) and approved by the Committee of Ethics of Biomedical Research of Andalusia (study code: 0679-N-21).

### Live-Cell Barcoding for Long-Term Drug Assays

Following live-cell barcoding, cells were cocultured for 24 h before the addition of doxorubicin (12.5 μM final) in complete media. After 6 and 24 h of doxorubicin exposure, cells were fixed, stained with antibodies, and processed for mass cytometry, as described.^10,52^ A gating strategy for selecting singlets (total cell population) was carried out (Figure S1).

### Statistical Analysis

Each experiment was performed in triplicate. Data sets are presented as mean ± standard deviation. Statistical significance was determined using multiple comparisons by a two-way analysis of variance (ANOVA). *p*-values ≤ 0.05 were considered significant. GraphPad Prism 8.0 (GraphPad Software Inc.) was used for graph plotting and statistics.

## Results and Discussion

### Preparation and Characterization of Hybrid-Tag Nanotrackers

**Figure 1:**
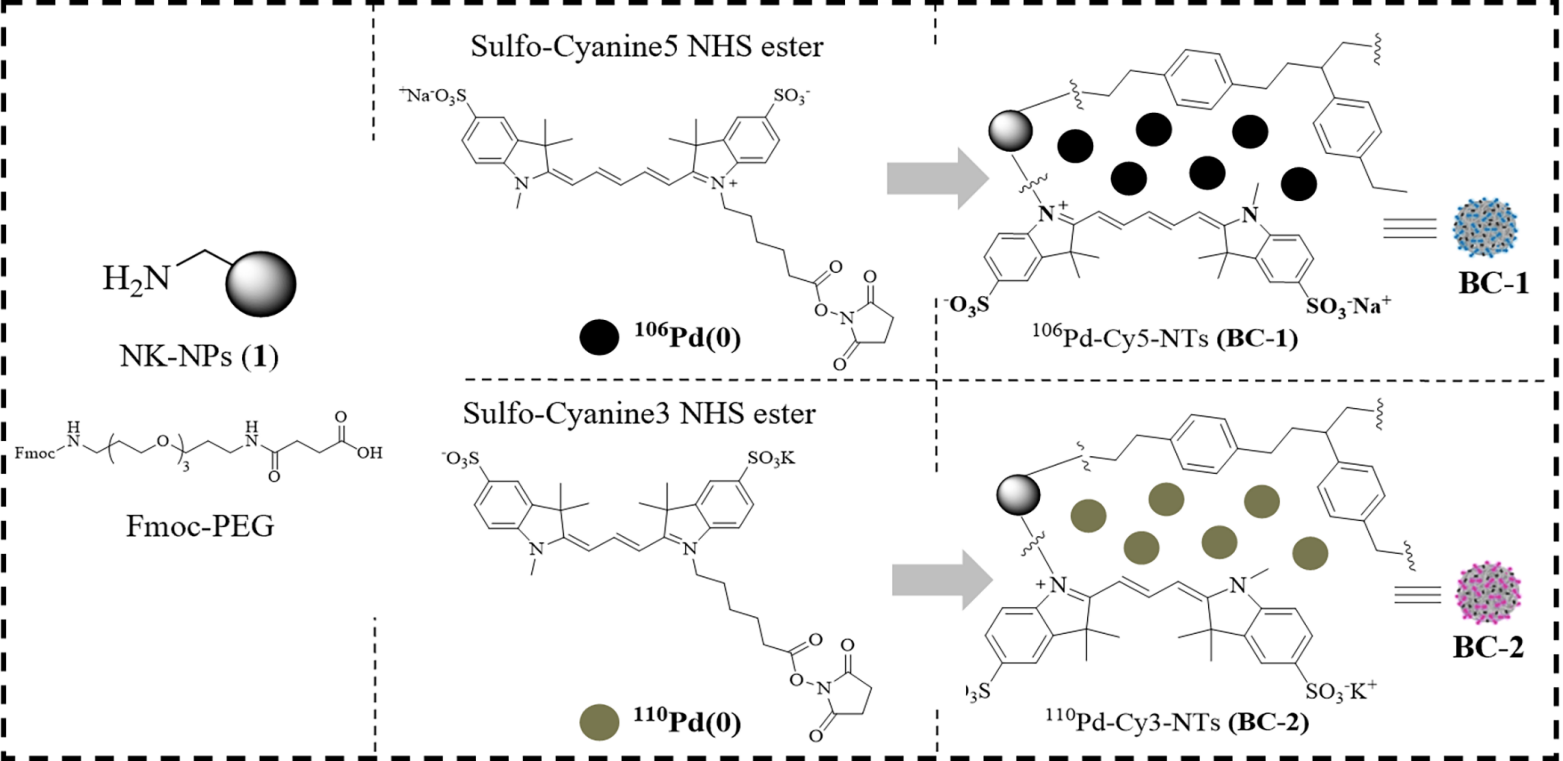
General scheme for hybrid-tag nanotracker preparation.

For proof of concept, two different barcoded polystyrene hybrid-tag nanotrackers carrying different palladium isotopes and fluorophores were produced using a previously reported solid-phase chemistry protocol.^29^ Briefly, a Fmoc-protected poly(ethylene glycol) (Fmoc-PEG) spacer was conjugated to amino-functionalized cross-linked polystyrene nanoparticles, as previously reported.^51^ After Fmoc deprotection, a cyanine fluorophore (Cy3 or Cy5) was conjugated to PEGylated NPs. In the next step, isotopically enriched palladium ^106^Pd and ^110^Pd were coordinated to the electron-rich network formed between the cyanine polymethine chain and the polystyrene aromatic rings. Two different hybrid-tag nanotrackers, ^106^Pd-Cy5-NTs (**Barcode-1**, **BC-1**) and ^110^Pd-Cy3-NTs (**Barcode-2**, **BC-2**), were obtained (Figure 1) after the reduction of Pd(II) to Pd(0). A detailed synthetic scheme is shown in the SI. Two additional hybrid-tag nanotrackers (**BC-S1** and **BC-S2**) were produced by recombination of the metal and fluorophore labels to prove the robustness and reproducibility of this protocol (Scheme S1). Importantly, the versatility of this protocol is not limited to the palladium isotopes and cyanine fluorophores reported here but has versatility toward other metals and fluorophores.

**Figure 2:**
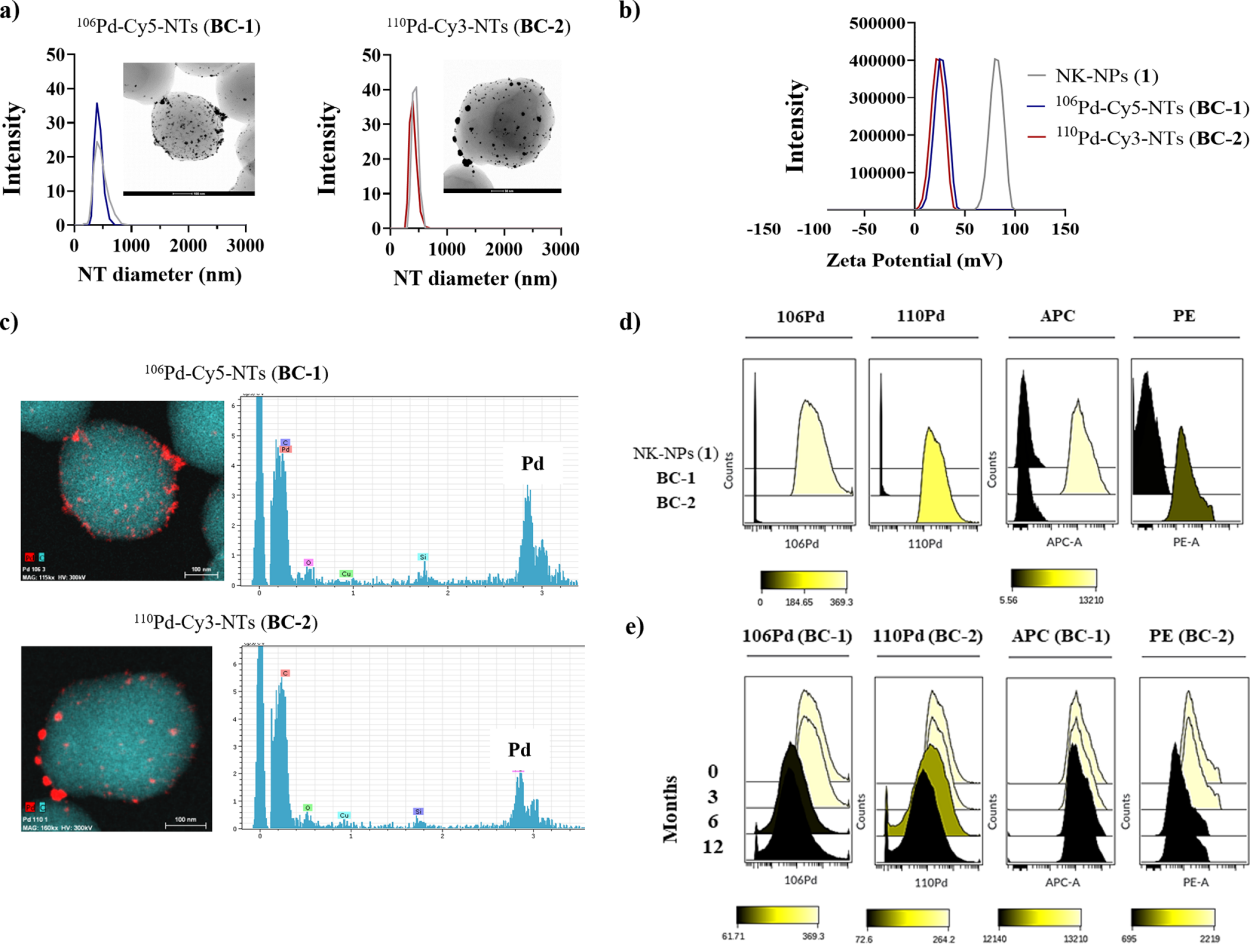
Physical–chemical characterization of hybrid-tag nanotrackers ^106^Pd-Cy5-NTs (**BC-1**) and ^110^Pd-Cy3-NTs (**BC-2**). (a) Histograms show hydrodynamic diameter values of hybrid-tag nanotrackers with and without (gray) Pd, determined by DLS, of **BC-1** (left) and **BC-2** (right) nanotrackers. Inset images are representative TEM images of the corresponding hybrid-tag nanotracker; (b) ζ-potential values; (c) EDX-HRTEM images and EDX analysis of **BC-1** (top) and **BC-2** (bottom) of the Pd signal from the developed hybrid-tag nanotrackers (carbon signal in blue, palladium signal in red); (d) histograms of Pd and fluorophore channels measured by mass (left) and flow cytometry (right). APC channel for Cy5 and PE for Cy3; (e) signal of Pd isotopes and fluorophores conjugated to hybrid-tag nanotrackers after 0, 3, 6, and 12 months of **BC-1** and **BC-2** measured by mass (left) and flow cytometry (right). Freshly prepared nanotrackers were used as control (0 months).

The physicochemical characterization of the polymeric hybrid-tag nanotrackers ^106^Pd-Cy5-NTs (**BC-1**) and ^110^Pd-Cy3-NTs (**BC-2**) was performed by DLS, HRTEM, XPS, and mass cytometry (Figure 2). As a control, we used a non-conjugated polystyrene nanoparticle (NK-NPs (**1**)). The hydrodynamic size of NK-NPs (**1**) and hybrid-tag nanotrackers was measured by DLS, being 410.3 nm with a polydispersity index (PDI) of 0.095 for NK-NPs (**1**), 412.6 nm and a PDI of 0.109 for **BC-1**, and 401.5 nm and a PDI of 0.075 for **BC-2** (Figure 2a). These results show that these nanoparticles are monodisperse populations and that the labeling with Pd and fluorophore does not affect their monodispersity. ζ-Potential values were +25.4 and +21.3 mV for **BC-1** and **BC-2** (Figure 2b), respectively. Morphology of the hybrid-tag nanotrackers was analyzed by HRTEM which confirmed their characteristic spherical shape (Figure 2a, insets). The effective incorporation of Pd isotopes was evaluated by EDX analysis (Figure 2c) and XPS (Figure S2a). Pd and fluorescence signals of hybrid-tag nanotrackers were successfully detected by mass and flow cytometry (Figure 2d). Noteworthy was the absence of any spillover between the ^106^Pd and ^110^Pd channels in mass cytometry. Despite a slight drop in the Pd signal observed overtime, the stability and robustness of the hybrid-tag nanotrackers, stored in water at 4 °C for 12 months, were proven by mass and flow cytometry (Figure 2e). A complete characterization of additional hybrid-tag nanotrackers (**BC-S1** and **BC-S2**) is shown in the SI (Figures S2b and S3).

### Long-Term Stability and Cytotoxicity of Intracellular Hybrid-Tag Nanotrackers

**Figure 3:**
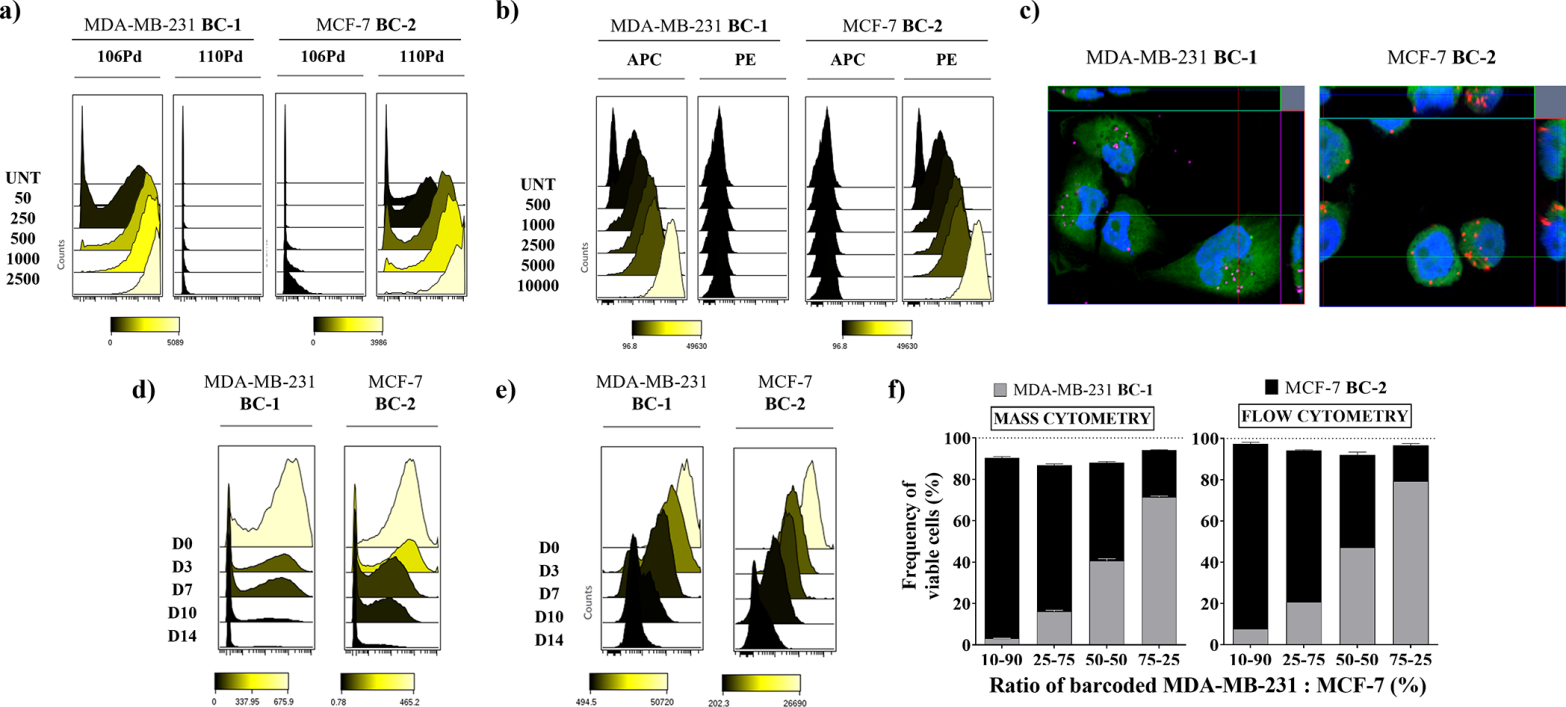
Cell assays performed with ^106^Pd-Cy5-NTs (**BC-1**) and ^110^Pd-Cy3-NTs (**BC-2**) after 3 h of incubation in MDA-MB-231 and MCF-7 breast cancer cell lines, respectively. (a) Cellular uptake of hybrid-tag nanotrackers **BC-1** and **BC-2** analyzed by mass cytometry; (b) cellular uptake of hybrid-tag nanotrackers **BC-1** and **BC-2** analyzed by flow cytometry. APC channel for Cy5 and PE for Cy3; (c) orthogonal views (*xy, xz*, and *yz* planes) of representative confocal microscopy images showing intersection planes at cross-line positions. MDA-MB-231 barcoded with **BC-1** (left) and MCF-7 cells with **BC-2** (right). The cytoplasm was stained using CellTracker Green, and the nuclei were stained with DAPI (blue); (d) palladium mass signal monitored by mass cytometry for a time course (days 3, 7, 10, and 14) after nanotracker incubation; (e) fluorescent signal monitored by flow cytometry for a time course (days 3, 7, 10, and 14) after nanotracker incubation; and (f) frequency of viable cells analyzed from cocultures by mass cytometry (left) and flow cytometry (right).

Many studies have confirmed that polymeric nanoparticles are readily internalized by a wide variety of cells with no significant signs of toxicity.^29,44−47,49,50,53−55^ Internalization efficiency and cytotoxicity of all prepared hybrid-tag nanotrackers were assessed in two breast cancer cell lines, MDA-MB-231 and MCF-7, by mass and flow cytometry (details in the SI). For that, cells were incubated with hybrid-tag nanotrackers ^106^Pd-Cy5-NTs (**BC-1**) and ^110^Pd-Cy3-NTs (**BC-2**), at different concentrations of NTs/cell (from 50 to 2,500) at RT for 3 h. The gating strategy used for the analysis is specified in Figure S1. The internalization of hybrid-tag nanotrackers was measured by quantifying the Pd-mass signal by mass cytometry. Hybrid-tag nanotrackers **BC-1** and **BC-2** showed similar uptake efficiency in MDA-MB-231 and MCF-7 cell lines by mass cytometry (Figures 3 and S5a,b). Additional hybrid-tag nanotrackers **BC-S1** and **BC-S2** showed similar behavior (Figure S5a,b). An internalization efficiency of 100% was obtained at 2,500 NTs/cell in both cell lines (Figures 3a and S5a,b). The dual readout of nanotrackers (mass and fluorescence) enabled the analysis of hybrid-tag nanotrackers by traditional fluorescence-based flow cytometry. Cellular uptake evaluated by flow cytometry showed similar results to those obtained by mass cytometry (Figures 3b and S5c,d), demonstrating the compatibility of these hybrid-tag nanotrackers with fluorescent techniques such as flow cytometry. We additionally confirmed the internalization of the hybrid-tag reagents by confocal microscopy. Z-stack images showed that nanotrackers were inside cells (Figure 3c). These results are consistent with published studies from our and other groups about the cellular uptake of polystyrene nanoparticles by many types of cells: adherent, suspension, stem, and primary.^56,57^ To assess the duration of hybrid-tag nanotracker cellular internalization, we used optimized conditions (2,500 NTs/cell for 3 h) to barcode MDA-MB-231 and MCF-7 cell lines with ^106^Pd-Cy5-NTs (**BC-1**) and ^110^Pd-Cy3-NTs (**BC-2**), respectively. Then, we measured the Pd and fluorescence signals at different time points (0, 3, 7, 10, and 14 days) by mass and flow cytometry. Histograms of Pd-mass and fluorescence intensity corresponding to each hybrid-tag nanotracker are shown in Figure 3d,e.

Our results showed that the Pd-mass signal and fluorescence signal of barcoded cells could be detected for the entire time course (Table S1). However, over time, we observed a decrease in intensity due to cell division according to their known cell doubling time, as previously reported.^47^ We estimate that our nanotrackers could be traceable for 15–20 days.

To assess the cytotoxic properties of the hybrid-tag nanotrackers ^106^Pd-Cy5-NTs (**BC-1**) and ^110^Pd-Cy3-NTs (**BC-2**), MDA-MB-231 and MCF-7 cell lines were incubated with hybrid-tag nanotrackers (range from 156 to 20,000 NTs/cell) for 72 h. Cell viability was evaluated with the resazurin assay, and data were normalized with respect to untreated cells (100%). Even with a nanotracker concentration of 10-fold higher than that used in our experiments, we did not detect any cytotoxic effects (Figure S5e,f). We therefore conclude that these nanotrackers are innocuous, in accordance with our previously developed nanodevices.^29,44−47,49,50,53−55^

A critical attribute of barcoding reagents is that the signal should be stable inside barcoded cells over time when they are mixed in a coculture. Thus, we mixed MDA-MB-231 cells barcoded with ^106^Pd-Cy5-NTs (**BC-1**) and MCF-7 cells barcoded with ^110^Pd-Cy3-NTs (**BC-2**) at different ratios (10–90, 25–75, 50–50, and 75–25%). After 24 h of coculture, cells were harvested and processed for mass cytometry. Yields of viable cells were comparable to their starting numbers, which indicates that nanotrackers have nondetectable toxicity, confirming their suitability as reagents for live-cell barcoding (Figures 3f, left, and S6). In parallel, fluorescence-based flow cytometry confirmed that there was no intercellular transfer of the hybrid-tag nanotrackers (Figure 3f, right).

### Compatibility of Hybrid-Tag Nanotrackers for Live-Cell Barcoding with Mass Cytometry Sample Processing

The previous sections demonstrated that the nanotrackers are efficiently internalized and possess long-term stability in live cells, in addition to being innocuous. Our next experiments were designed to test the compatibility of nanotrackers with other mass cytometry reagents and ensure there would be no adverse effects on protein readouts. MDA-MB-231 and MCF-7 cell lines were individually barcoded with ^106^Pd-Cy5-NTs (**BC-1**) and ^110^Pd-Cy3-NTs (**BC-2**), respectively, and processed after 24 and 48 h in culture.

**Figure 4:**
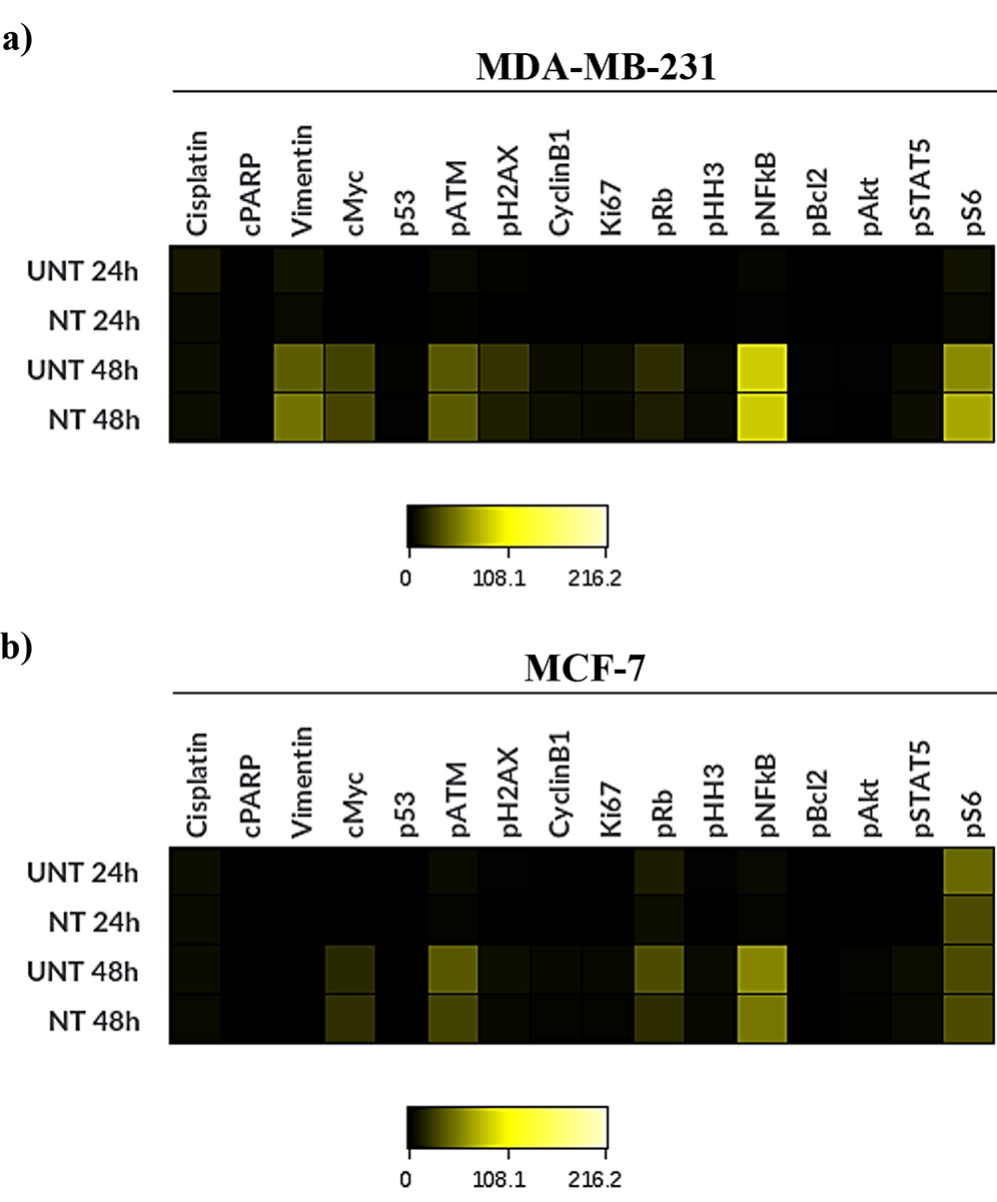
Heatmaps depict protein expression levels (median counts) of barcoded cell lines after 24 and 48 h of incubation with hybrid-tag nanotrackers by mass cytometry: (a) MDA-MB-231 cells barcoded with **BC-1** and (b) MCF-7 cells with **BC-2**.

Cells were stained with a previously validated mass cytometry antibody panel designed to measure intracellular protein expression levels in epithelial cancers.^10,52^ The panel included a set of different proteins according to their biological functions: (i) cancer-related proteins (vimentin, p53, and c-Myc) and (ii) cell cycle proteins (cyclin B1, pRb, and pHH3), apoptosis-related signaling pathways (pBCL-2, NF-κB), and STAT5 (Table S2). Cisplatin (Sigma-Aldrich) was used as a live–dead stain,^18^ and an antibody against cleaved PARP (cPARP) was used to quantify apoptotic cells (Figure S4). We observed that barcoded cell populations—channels ^106^Pd and ^110^Pd for MDA-MB-231 and MCF-7 cells, respectively—presented negligible platinum and cPARP levels and were similar to untreated cells. Notably, both proteomic profiles of barcoded cells and untreated cells were similar in both tested cell lines after 24 and 48 h, showing no significant alteration associated with cell damage (Figure 4).

These data reveal that the hybrid-tag nanotrackers do not interfere with the readout channels chosen for viability and metal-tagged antibodies used in this experiment. Nonrelevant changes in the expression of interrogated proteins are observed over time, independently of the barcode. No significant differences in protein expression are observed between control and barcoded cells. Based on these results, these hybrid-tag nanotrackers represent a new reagent that can be used for barcoding cells for long-term culture without any negative effects on experimental readouts in mass cytometry experiments.

Given that our barcoding nanosystem does not interfere with cellular proteins, fulfilling the main criteria for a universal cell barcoding, we believe that it could be compatible with other live-cell barcoding reagents, such as TeMal,^36^ platinum-labeled antibodies targeting b2m and CD298,^33^ and ratiometric barcoding with Pdots.^58^

### Assessing the Compatibility of Hybrid-Tag Nanotrackers on Heterogeneous Cell Populations by Flow and Mass Cytometry Dual-Modal Readout

Given that multiplexing studies are especially powerful on heterogeneous cell samples such as whole blood, further compatibility studies were carried out to evaluate the labeling efficiency of the hybrid-tag nanotrackers in non-cancer cells. Briefly, the whole blood sample was treated to isolate PBMCs and they were barcoded with hybrid-tag nanotrackers ^106^Pd-Cy5-NTs (**BC-1**) or ^110^Pd-Cy3-NTs (**BC-2**) prior to fluorescent antibody staining to sort them into monocytes (CD45+/CD14+) and T cells (CD45+/CD3+) barcoded with **BC-1** (Cy5+) and **BC-2** (Cy3+), respectively.

**Figure 5:**
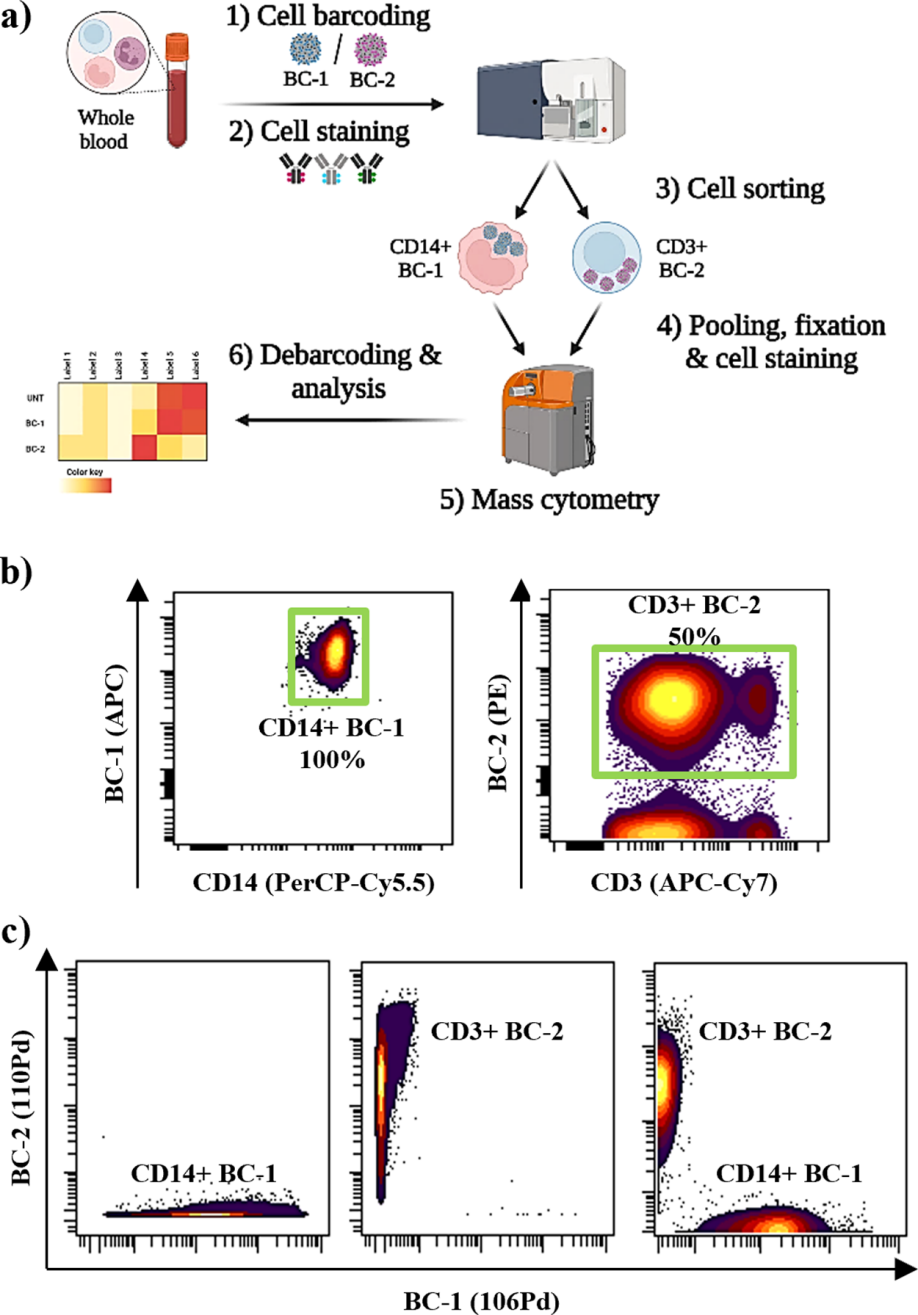
Compatibility of developed hybrid-tag nanotrackers with heterogeneous cell populations such as whole blood. (a) Schematic methodology to barcode different blood cell populations for flow and mass cytometry analysis; (b) evaluation of barcoding efficiency by fluorescence-activated cell sorting (FACS): dot plots of monocytes CD14+ (left), and T cells CD3+ (right) after the barcoding step. Barcoded cell populations were sorted (green gate); (c) evaluation of sorting efficiency by mass cytometry of **BC-1**/CD14+, **BC-2**/CD3+, and pooled barcoded populations.

Then, a mass cytometry analysis of a 50–50% pool of barcoded monocytes and T cells was performed, identifying each population according to their barcodes **BC-1** (106Pd+) and **BC-2** (110Pd+) (Figure 5a). Flow cytometry analysis shows a different degree of internalization based on the cell type, being 100 and 50% for monocytes and T cells, respectively. Interestingly, a higher intensity signal in barcoded monocytes compared to that in barcoded T cells can be observed, corroborating the different labeling efficiency in these two cell types (Figure 5b). Next, sorted barcoded monocytes and T cells were pooled and successfully tracked by mass cytometry thanks to the dual-modal signal of the hybrid-tag nanotrackers (Figure 5c). Toxicity assessment after nanotracker internalization showed no significant alteration associated with barcoding compared to cells without barcoding (Figure S7). These results confirm that our hybrid-tag nanotrackers are as suitable barcoding reagents for heterogeneous cell populations such as blood cells—as other reported mass cytometry reagents.^6,11,17,22,32,33^ However, a key differential aspect of our approach is that these hybrid-tag nanotrackers integrate both fluorescence and mass cytometry cell barcoding in a single device and allow a dual-modal readout. Based on these results, we can conclude that these hybrid-tag nanotrackers are suitable for barcoding of both heterogeneous and homogeneous cell populations. However, higher barcoding efficiency is achieved in homogeneous samples.

### Live-Cell Barcoding for Drug Assays

A series of experiments were carried out to evaluate the utility of hybrid-tag nanotrackers in cocultures of individually barcoded cells exposed to the chemotherapeutic agent doxorubicin.^59,60^ To select the optimal concentration of doxorubicin, we performed dose–response curves with MDA-MB-231 and MCF-7 cell lines (Figure S8). No damage was observed following 6 h of treatment measured by resazurin assay (Figure S8a). However, significant differences in doxorubicin potency were observed between the two cell lines when exposed to 12.5 μM of doxorubicin for 24 h. The viability was 30 and 70% for MCF-7 and MDA-MB-231 cell lines, respectively (Figure S8b).

**Figure 6:**
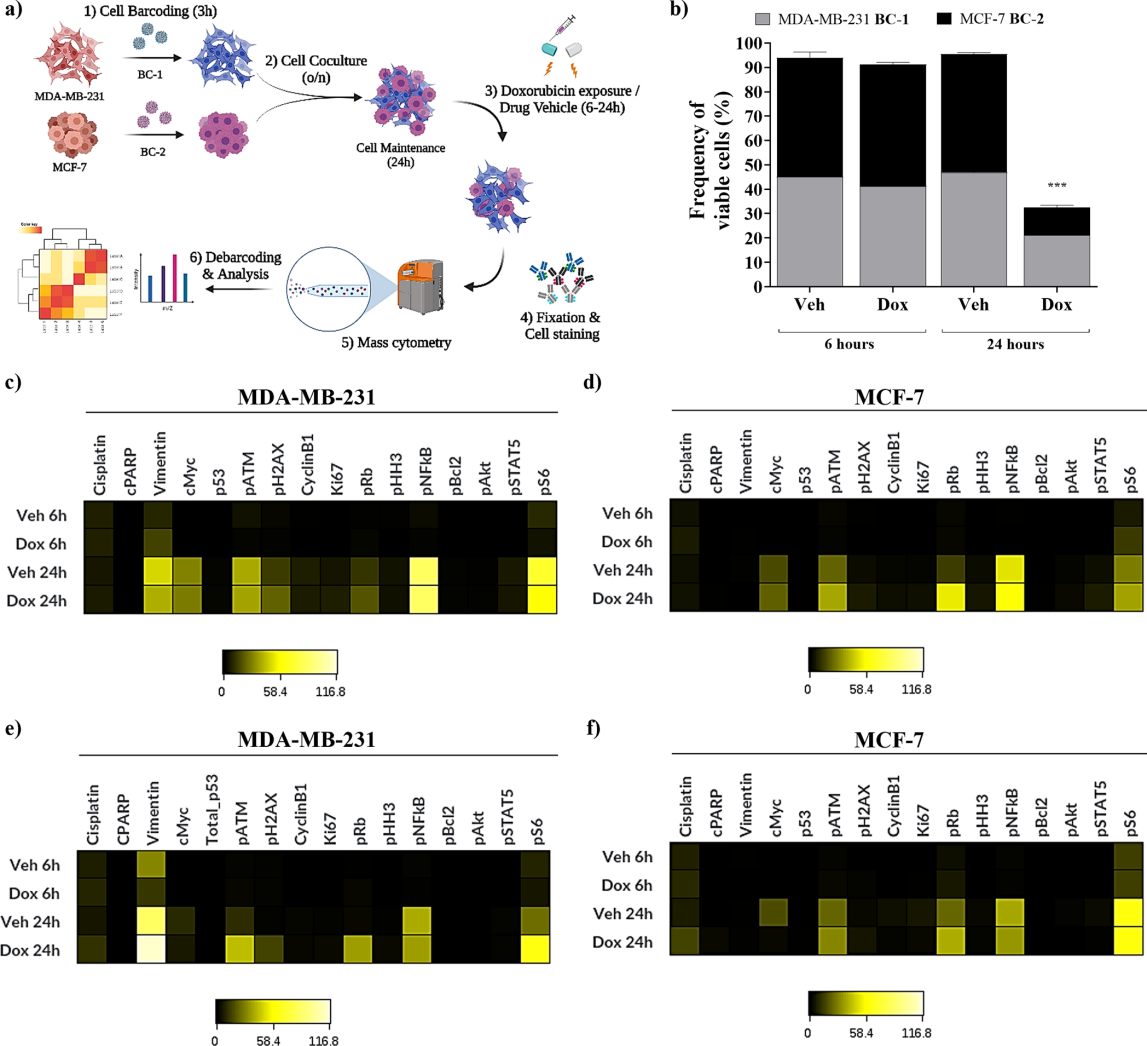
Tracking cell damage of barcoded cells in coculture after exposure to doxorubicin at 6 and 24 h by mass cytometry. (a) Schematic protocol for a cell-based drug assay using hybrid-tag nanotrackers; (b) frequencies of debarcoded cells recovered after doxorubicin exposure; (c) heatmap of protein expression levels in monocultured MDA-MB-231 and (d) MCF-7 debarcoded cells (raw median counts); and (e) heatmap of protein expression in cocultured debarcoded MDA-MB-231 and (f) MCF-7 cells.

We designed the experiment shown in Figure 6a. Briefly, we barcoded MDA-MB-231 cells with **BC-1** and MCF-7 cells with **BC-2** and cocultured them. Then, cells were treated with doxorubicin or sodium chloride (vehicle control) for 6 and 24 h. Cells were stained with an antibody panel against intracellular proteins and processed for mass cytometry (Table S2).

We determined the viability of each barcoded cell line by analyzing ^106^Pd or ^110^Pd -positive signals for MDA-MB-231 and MCF-7 cells, respectively. We observed that the number of viable MDA-MB-231 and MCF-7 cells following treatment with doxorubicin was approximately reduced by 60 and 76%, respectively, compared to cells treated with drug vehicle (untreated) (Figure 6b). Responses to doxorubicin (6 and 24 h) of each barcoded cell line in the coculture were comparable to those observed for each individually barcoded cell line (Figure S9). To complement the viability determination, we compared measurements of protein expression in coculture *versus* individually barcoded cells. Following debarcoding, expression levels of proteins expected to change in response to doxorubicin treatment were visualized on a heatmap. Our results showed concordance in the levels of the parameters reported in heatmaps of debarcoded cells with cells treated individually (Figure 6c–f). The expression patterns are similar in both monoculture and coculture for both cell lines, demonstrating that this approach does not interfere with the protein signature changes induced by drug treatment. Therefore, our method can be used to barcode live cells from the beginning of the assay, allowing monitoring of the cellular response to the drug treatment over time after debarcode is completed.

## Conclusions

In summary, we have developed fluorescent and mass hybrid-tag nanotrackers as new reagents that can be used for long-term live-cell barcoding and multiplexed drug assays and can be detected by flow and mass cytometry. These reagents have key advantages for cell barcoding such as fluorescence and mass dual-modality, durability (up to 14 days) without any appreciable cellular damage, stability over time, and universality since it does not interfere with cellular protein expression. Despite the use of only two hybrid-tag nanotrackers, this proof-of-principle barcoding strategy could be extended using other metal isotopes as tags.

Successful proteomic profiling of single cells was achieved with a 14-antibody panel in the presence of the hybrid-tag nanotrackers. These nanotrackers provide robust barcoding of live cells compatible with existing mass cytometry reagents (metal-labeled antibodies, DNA intercalators, and cisplatin) and cell processing protocols for mass cytometry, allowing the barcoding of heterogeneous cells together with flow and mass cytometry dual-modal readout.

This barcoding approach does not rely on proteins; therefore, it is not affected when proteomic changes are induced after exogenous perturbations, *e.g.*, drug treatments. Simultaneous measurements of cocultures with cells barcoded with nanotrackers over time following doxorubicin treatment have been successfully performed. Notably, the hybrid-tag nanotrackers have a significant advantage over other barcoding reagents in that they can be used for drug-sensitive and/or minority cell populations used in cocultures, avoiding harsh treatments or conditions that could mask cells under low signal intensities close to background. To the best of our knowledge, this is the first dual fluorescent and mass cytometry barcoding reagent compatible with long-term live-cell barcoding and multiplexed drug assays. In addition, these hybrid nanotrackers are also compatible with fluorescence-based techniques, such as confocal microscopy, opening the range of applications of this barcoding method.

## Supporting Information Available

Additional experimental details, materials, and methods for the development and evaluation of hybrid-tag nanotrackers (PDF)

## References

[ref1] Zunder E. R.; Finck R.; Behbehani G. K.; Amir E. A. D.; Krishnaswamy S.; Gonzalez V. D.; Lorang C. G.; Bjornson Z.; Spitzer M. H.; Bodenmiller B.; Fantl W. J.; Pe’Er D.; Nolan G. P. Palladium-Based Mass Tag Cell Barcoding with a Doublet-Filtering Scheme and Single-Cell Deconvolution Algorithm. Nat. Protoc. 2015, 10, 316–333. 10.1038/nprot.2015.020. 25612231 PMC4347881

[ref2] Reisman B. J.; Barone S. M.; Bachmann B. O.; Irish J. M. DebarcodeR Increases Fluorescent Cell Barcoding Capacity and Accuracy. Cytometry, Part A 2021, 99, 946–953. 10.1002/cyto.a.24363. PMC8410645 33960644

[ref3] Giudice V.; Feng X.; Kajigaya S.; Young N. S.; Biancotto A. Optimization and Standardization of Fluorescent Cell Barcoding for Multiplexed Flow Cytometric Phenotyping. Cytometry, Part A 2017, 91, 694–703. 10.1002/cyto.a.23162. PMC5612408 28692789

[ref4] Krutzik P. O.; Clutter M. R.; Trejo A.; Nolan G. P. Fluorescent Cell Barcoding for Multiplex Flow Cytometry. Curr. Protoc. Cytom. 2011, 55, 6.31.1–6.31.15. 10.1002/0471142956.cy0631s55. PMC3036011 21207359

[ref5] Bendall S. C.; Simonds E. F.; Qiu P.; Amir E. A. D.; Krutzik P. O.; Finck R.; Bruggner R. V.; Melamed R.; Trejo A.; Ornatsky O. I.; Balderas R. S.; Plevritis S. K.; Sachs K.; Pe’er D.; Tanner S. D.; Nolan G. P. Single-Cell Mass Cytometry of Differential Immune and Drug Responses across a Human Hematopoietic Continuum. Science 2011, 332, 687–696. 10.1126/science.1198704. 21551058 PMC3273988

[ref6] Bjornson Z. B.; Nolan G. P.; Fantl W. J. Single Cell Mass Cytometry for Analysis of Immune System Functional States. Curr. Opin. Immunol. 2013, 25, 484–494. 10.1016/j.coi.2013.07.004. 23999316 PMC3835664

[ref7] Spitzer M. H.; Nolan G. P. Mass Cytometry: Single Cells, Many Features. Cell 2016, 165, 780–791. 10.1016/j.cell.2016.04.019. 27153492 PMC4860251

[ref8] Bandura D. R.; Baranov V. I.; Ornatsky O. I.; Antonov A.; Kinach R.; Lou X.; Pavlov S.; Vorobiev S.; Dick J. E.; Tanner S. D. Mass Cytometry: Technique for Real Time Single Cell Multitarget Immunoassay Based on Inductively Coupled Plasma Time-of-Flight Mass Spectrometry. Anal. Chem. 2009, 81, 6813–6822. 10.1021/ac901049w. 19601617

[ref9] Harmsen S.; Coskun A. F.; Ganesh S.; Nolan G. P.; Gambhir S. S. Isotopically Encoded Nanotags for Multiplexed Ion Beam Imaging. Adv. Mater. Technol. 2020, 5, 2000098 10.1002/admt.202000098. 32661501 PMC7357881

[ref10] Gonzalez V. D.; Huang Y. W.; Delgado-Gonzalez A.; Chen S. Y.; Donoso K.; Sachs K.; Gentles A. J.; Allard G. M.; Kolahi K. S.; Howitt B. E.; Porpiglia E.; Fantl W. J. High-Grade Serous Ovarian Tumor Cells Modulate NK Cell Function to Create an Immune-Tolerant Microenvironment. Cell Rep. 2021, 36, 109632 10.1016/j.celrep.2021.109632. 34469729 PMC8546503

[ref11] Han G.; Spitzer M. H.; Bendall S. C.; Fantl W. J.; Nolan G. P. Metal-Isotope-Tagged Monoclonal Antibodies for High-Dimensional Mass Cytometry. Nat. Protoc. 2018, 13, 2121–2148. 10.1038/s41596-018-0016-7. 30258176 PMC7075473

[ref12] Zhang Y.; Zabinyakov N.; Majonis D.; Bouzekri A.; Ornatsky O.; Baranov V.; Winnik M. A. Tantalum Oxide Nanoparticle-Based Mass Tag for Mass Cytometry. Anal. Chem. 2020, 92, 5741–5749. 10.1021/acs.analchem.9b04970. 32239915

[ref13] Liu J.; Jarzabek J.; Majonis D.; Watson J.; Baranov V.; Winnik M. A. Metal-Encoded Polystyrene Microbeads as a Mass Cytometry Calibration/Normalization Standard Covering Channels from Yttrium (89 Amu) to Bismuth (209 Amu). Anal. Chem. 2020, 92, 999–1006. 10.1021/acs.analchem.9b03935. 31815445

[ref14] Lou X.; Zhang G.; Herrera I.; Kinach R.; Ornatsky O.; Baranov V.; Nitz M.; Winnik M. A. Polymer-Based Elemental Tags for Sensitive Bioassays. Angew. Chem., Int. Ed. 2007, 46, 6111–6114. 10.1002/anie.200700796. PMC2504858 17533637

[ref15] Cho H.; Liu P.; Pichaandi J.; Closson T. L. L.; Majonis D.; Leighton P. L. A.; Swanson E.; Ornatsky O.; Baranov V.; Winnik M. A. A Metal-Chelating Polymer for Chelating Zirconium and Its Use in Mass Cytometry. Eur. Polym. J. 2019, 120, 109175 10.1016/j.eurpolymj.2019.08.002.

[ref16] Ornatsky O. I.; Lou X.; Nitz M.; Schäfer S.; Sheldrick W. S.; Baranov V. I.; Bandura D. R.; Tanner S. D. Study of Cell Antigens and Intracellular DNA by Identification of Element-Containing Labels and Metallointercalators Using Inductively Coupled Plasma Mass Spectrometry. Anal. Chem. 2008, 80, 2539–2547. 10.1021/ac702128m. 18318509

[ref17] Behbehani G. K.; Bendall S. C.; Clutter M. R.; Fantl W. J.; Nolan G. P. Single-Cell Mass Cytometry Adapted to Measurements of the Cell Cycle. Cytometry, Part A 2012, 81 A, 552–566. 10.1002/cyto.a.22075. PMC3667754 22693166

[ref18] Fienberg H. G.; Simonds E. F.; Fantl W. J.; Nolan G. P.; Bodenmiller B. A Platinum-Based Covalent Viability Reagent for Single-Cell Mass Cytometry. Cytometry, Part A 2012, 81 A, 467–475. 10.1002/cyto.a.22067. PMC3808967 22577098

[ref19] Majonis D.; Ornatsky O.; Kinach R.; Winnik M. A. Curious Results with Palladium- and Platinum-Carrying Polymers in Mass Cytometry Bioassays and an Unexpected Application as a Dead Cell Stain. Biomacromolecules 2011, 12, 3997–4010. 10.1021/bm201011t. 21955116

[ref20] Park H.; Edgar L. J.; Lumba M. A.; Willis L. M.; Nitz M. Organotellurium Scaffolds for Mass Cytometry Reagent Development. Org. Biomol. Chem. 2015, 13, 7027–7033. 10.1039/C5OB00593K. 26040785

[ref21] Behbehani G. K.; Thom C.; Zunder E. R.; Finck R.; Gaudilliere B.; Fragiadakis G. K.; Fantl W. J.; Nolan G. P. Transient Partial Permeabilization with Saponin Enables Cellular Barcoding Prior to Surface Marker Staining. Cytometry, Part A 2014, 85, 1011–1019. 10.1002/cyto.a.22573. PMC4361015 25274027

[ref22] Mei H. E.; Leipold M. D.; Maecker H. T. Platinum-Conjugated Antibodies for Application in Mass Cytometry. Cytometry, Part A 2016, 89, 292–300. 10.1002/cyto.a.22778. 26355391

[ref23] Wu X.; DeGottardi Q.; Wu I. C.; Yu J.; Wu L.; Ye F.; Kuo C. T.; Kwok W. W.; Chiu D. T. Lanthanide-Coordinated Semiconducting Polymer Dots Used for Flow Cytometry and Mass Cytometry. Angew. Chem., Int. Ed. 2017, 56, 14908–14912. 10.1002/anie.201708463. PMC5687989 28941061

[ref24] Ha M. K.; Kwon S. J.; Choi J. S.; Nguyen N. T.; Song J.; Lee Y.; Kim Y. E.; Shin I.; Nam J. W.; Yoon T. H. Mass Cytometry and Single-Cell RNA-Seq Profiling of the Heterogeneity in Human Peripheral Blood Mononuclear Cells Interacting with Silver Nanoparticles. Small 2020, 16, 1907674 10.1002/smll.201907674. 32163679

[ref25] Pichaandi J.; Zhao G.; Bouzekri A.; Lu E.; Ornatsky O.; Baranov V.; Nitz M.; Winnik M. A. Lanthanide Nanoparticles for High Sensitivity Multiparameter Single Cell Analysis. Chem. Sci. 2019, 10, 2965–2974. 10.1039/C8SC04407D. 30996875 PMC6427950

[ref26] Schulz A. R.; Stanislawiak S.; Baumgart S.; Grützkau A.; Mei H. E. Silver Nanoparticles for the Detection of Cell Surface Antigens in Mass Cytometry. Cytometry, Part A 2017, 91, 25–33. 10.1002/cyto.a.22904. 27351740

[ref27] Yang Y. S. S.; Atukorale P. U.; Moynihan K. D.; Bekdemir A.; Rakhra K.; Tang L.; Stellacci F.; Irvine D. J. High-Throughput Quantitation of Inorganic Nanoparticle Biodistribution at the Single-Cell Level Using Mass Cytometry. Nat. Commun. 2017, 8, 14069 10.1038/ncomms14069. 28094297 PMC5247578

[ref28] Abdelrahman A. I.; Dai S.; Thickett S. C.; Ornatsky O.; Bandura D.; Baranov V.; Winnik M. A. Lanthanide-Containing Polymer Microspheres by Multiple-Stage Dispersion Polymerization for Highly Multiplexed Bioassays. J. Am. Chem. Soc. 2009, 131, 15276–15283. 10.1021/ja9052009. 19807075 PMC2801425

[ref29] Delgado-Gonzalez A.; Garcia-Fernandez E.; Valero T.; Victoria Cano-Cortes M.; Ruedas-Rama M. J.; Unciti-Broceta A.; Sanchez-Martin R. M.; Diaz-Mochon J. J.; Orte A. Metallofluorescent Nanoparticles for Multimodal Applications. ACS Omega 2018, 3, 144–153. 10.1021/acsomega.7b01984. 30023770 PMC6044963

[ref30] Bodenmiller B.; Zunder E. R.; Finck R.; Chen T. J.; Savig E. S.; Bruggner R. V.; Simonds E. F.; Bendall S. C.; Sachs K.; Krutzik P. O.; Nolan G. P. Multiplexed Mass Cytometry Profiling of Cellular States Perturbed by Small-Molecule Regulators. Nat. Biotechnol. 2012, 30, 858–867. 10.1038/nbt.2317. 22902532 PMC3627543

[ref31] Mei H. E.; Leipold M. D.; Schulz A. R.; Chester C.; Maecker H. T. Barcoding of Live Human Peripheral Blood Mononuclear Cells for Multiplexed Mass Cytometry. J. Immunol. 2015, 194, 2022–2031. 10.4049/jimmunol.1402661. 25609839 PMC4323739

[ref32] Muftuoglu M.; Li L.; Liang S.; Mak D.; Lin A. J.; Fang J.; Burks J. K.; Chen K.; Andreeff M. Extended Live-Cell Barcoding Approach for Multiplexed Mass Cytometry. Sci. Rep. 2021, 11, 12388 10.1038/s41598-021-91816-w. 34117319 PMC8196040

[ref33] Hartmann F. J.; Simonds E. F.; Bendall S. C. A Universal Live Cell Barcoding-Platform for Multiplexed Human Single Cell Analysis. Sci. Rep. 2018, 8, 10770 10.1038/s41598-018-28791-2. 30018331 PMC6050312

[ref34] Charmsaz S.; Gross N.; Jaffee E.; Ho W. J. A Global Live Cell Barcoding Approach for Multiplexed Mass Cytometry Profiling of Mouse Tumors. JCI Insight 2021, 6, e143283 10.1172/jci.insight.143283. PMC8119183 33690223

[ref35] Catena R.; Özcan A.; Zivanovic N.; Bodenmiller B. Enhanced Multiplexing in Mass Cytometry Using Osmium and Ruthenium Tetroxide Species. Cytometry, Part A 2016, 89, 491–497. 10.1002/cyto.a.22848. 27018769

[ref36] Willis L. M.; Park H.; Watson M. W. L.; Majonis D.; Watson J. L.; Nitz M. Tellurium-Based Mass Cytometry Barcode for Live and Fixed Cells. Cytometry, Part A 2018, 93, 685–694. 10.1002/cyto.a.23495. 30053343

[ref37] Qin X.; Sufi J.; Vlckova P.; Kyriakidou P.; Acton S. E.; Li V. S. W.; Nitz M.; Tape C. J. Cell-Type-Specific Signaling Networks in Heterocellular Organoids. Nat. Methods 2020, 17, 335–342. 10.1038/s41592-020-0737-8. 32066960 PMC7060080

[ref38] Sufi J.; Qin X.; Rodriguez F. C.; Bu Y. J.; Vlckova P.; Zapatero M. R.; Nitz M.; Tape C. J. Multiplexed Single-Cell Analysis of Organoid Signaling Networks. Nat. Protoc. 2021, 16, 4897–4918. 10.1038/s41596-021-00603-4. 34497385

[ref39] Teh C. E.; Gong J. N.; Segal D.; Tan T.; Vandenberg C. J.; Fedele P. L.; Low M. S. Y.; Grigoriadis G.; Harrison S. J.; Strasser A.; Roberts A. W.; Huang D. C. S.; Nolan G. P.; Gray D. H. D.; Ko M. E. Deep Profiling of Apoptotic Pathways with Mass Cytometry Identifies a Synergistic Drug Combination for Killing Myeloma Cells. Cell Death Differ. 2020, 27, 2217–2233. 10.1038/s41418-020-0498-z. 31988495 PMC7308383

[ref40] Georgopoulou D.; Callari M.; Rueda O. M.; et al. Landscapes of Cellular Phenotypic Diversity in Breast Cancer Xenografts and Their Impact on Drug Response. Nat. Commun. 2021, 12, 1998 10.1038/s41467-021-22303-z. 33790302 PMC8012607

[ref41] Majonis D.; Ornatsky O.; Weinrich D.; Winnik M. A. Dual-Purpose Polymer Labels for Fluorescent and Mass Cytometric Affinity Bioassays. Biomacromolecules 2013, 14, 1503–1513. 10.1021/bm4001662. 23574014

[ref42] Xu H.; Zhang Z.; Wang Y.; Zhang X.; Zhu J.-J.; Min Q. Sense and Validate: Fluorophore/Mass Dual-Encoded Nanoprobes for Fluorescence Imaging and MS Quantification of Intracellular Multiple MicroRNAs. Anal. Chem. 2022, 94, 6329–6337. 10.1021/acs.analchem.2c00513. 35412806

[ref43] Pich A.; Zhang F.; Shen L.; Berger S.; Ornatsky O.; Baranov V.; Winnik M. A. Biocompatible Hybrid Nanogels**. Small 2008, 4, 2171–2175. 10.1002/smll.200801159. 19003827 PMC2766817

[ref44] Sanchez-Martin R. M.; Muzerelle M.; Chitkul N.; How S. E.; Mittoo S.; Bradley M. Bead-Based Cellular Analysis, Sorting and Multiplexing. ChemBioChem 2005, 6, 1341–1345. 10.1002/cbic.200500059. 15973759

[ref45] Cano-Cortes M. V.; Navarro-Marchal S. A.; Ruiz-Blas M. P.; Diaz-Mochon J. J.; Marchal J. A.; Sanchez-Martin R. M. A Versatile Theranostic Nanodevice Based on an Orthogonal Bioconjugation Strategy for Efficient Targeted Treatment and Monitoring of Triple Negative Breast Cancer. Nanomedicine 2020, 24, 102120 10.1016/j.nano.2019.102120. 31676374

[ref46] Bradley M.; Alexander L.; Duncan K.; Chennaoui M.; Jones A. C.; Sánchez-Martín R. M. PH Sensing in Living Cells Using Fluorescent Microspheres. Bioorg. Med. Chem. Lett. 2008, 18, 313–317. 10.1016/j.bmcl.2007.10.075. 17988866

[ref47] Altea-Manzano P.; Unciti-Broceta J. D.; Cano-Cortes V.; Ruiz-Blas M. P.; Valero-Griñan T.; Diaz-Mochon J. J.; Sanchez-Martin R. Tracking Cell Proliferation Using a Nanotechnology-Based Approach. Nanomedicine 2017, 12, 1591–1605. 10.2217/nnm-2017-0118. 28513331

[ref48] Valero T.; Delgado-González A.; Unciti-Broceta J. D.; Cano-Cortés V.; Pérez-López A. M.; Unciti-Broceta A.; Sánchez Martín R. M. Drug “Clicking” on Cell-Penetrating Fluorescent Nanoparticles for in Cellulo Chemical Proteomics. Bioconjugate Chem. 2018, 29, 3154–3160. 10.1021/acs.bioconjchem.8b00481. 30122043

[ref49] Cano-Cortes M. V.; Altea-Manzano P.; Laz-Ruiz J. A.; Unciti-Broceta J. D.; Lopez-Delgado F. J.; Espejo-Roman J. M.; Diaz-Mochon J. J.; Sanchez-Martin R. M. An Effective Polymeric Nanocarrier That Allows for Active Targeting and Selective Drug Delivery in Cell Coculture Systems. Nanoscale 2021, 13, 3500–3511. 10.1039/D0NR07145E. 33560282

[ref50] Yusop R. M.; Unciti-Broceta A.; Johansson E. M. V.; Sánchez-Martín R. M.; Bradley M. Palladium-Mediated Intracellular Chemistry. Nat. Chem. 2011, 3, 239–243. 10.1038/nchem.981. 21336331

[ref51] Unciti-Broceta A.; Johansson E. M. V.; Yusop R. M.; Sánchez-Martín R. M.; Bradley M. Synthesis of Polystyrene Microspheres and Functionalization with Pd0 Nanoparticles to Perform Bioorthogonal Organometallic Chemistry in Living Cells. Nat. Protoc. 2012, 7, 1207–1218. 10.1038/nprot.2012.052. 22653159

[ref52] Gonzalez V. D.; Samusik N.; Chen T. J.; Savig E. S.; Aghaeepour N.; Quigley D. A.; Huang Y. W.; Giangarrà V.; Borowsky A. D.; Hubbard N. E.; Chen S. Y.; Han G.; Ashworth A.; Kipps T. J.; Berek J. S.; Nolan G. P.; Fantl W. J. Commonly Occurring Cell Subsets in High-Grade Serous Ovarian Tumors Identified by Single-Cell Mass Cytometry. Cell Rep. 2018, 22, 1875–1888. 10.1016/j.celrep.2018.01.053. 29444438 PMC8556706

[ref53] Alexander L. M.; Pernagallo S.; Livigni A.; Sánchez-Martín R. M.; Brickman J. M.; Bradley M. Investigation of Microsphere-Mediated Cellular Delivery by Chemical, Microscopic and Gene Expression Analysis. Mol. Biosyst. 2010, 6, 399–409. 10.1039/B914428E. 20094660

[ref54] Cano-Cortes M. V.; Laz-Ruiz J. A.; Diaz-Mochon J. J.; Sanchez-Martin R. M. Characterization and Therapeutic Effect of a PH Stimuli Responsive Polymeric Nanoformulation for Controlled Drug Release. Polymers 2020, 12, 1265 10.3390/polym12061265. PMC7361709 32492910

[ref55] Unciti-Broceta J. D.; Cano-Cortés V.; Altea-Manzano P.; Pernagallo S.; Díaz-Mochón J. J.; Sánchez-Martín R. M. Number of Nanoparticles per Cell through a Spectrophotometric Method - A Key Parameter to Assess Nanoparticle-Based Cellular Assays. Sci. Rep. 2015, 5, 10091 10.1038/srep10091. 25976173 PMC4432369

[ref56] Gennet N.; Alexander L. M.; Sánchez-Martín R. M.; Behrendt J. M.; Sutherland A. J.; Brickman J. M.; Bradley M.; Li M. Microspheres as a Vehicle for Biomolecule Delivery to Neural Stem Cells. New Biotechnol. 2009, 25, 442–449. 10.1016/j.nbt.2009.05.006. 19524076

[ref57] Tsakiridis A.; Alexander L. M.; Gennet N.; Sanchez-Martin R. M.; Livigni A.; Li M.; Bradley M.; Brickman J. M. Microsphere-Based Tracing and Molecular Delivery in Embryonic Stem Cells. Biomaterials 2009, 30, 5853–5861. 10.1016/j.biomaterials.2009.06.024. 19608269

[ref58] Wu X.; Degottardi Q.; Wu I. C.; Wu L.; Yu J.; Kwok W. W.; Chiu D. T. Ratiometric Barcoding for Mass Cytometry. Anal. Chem. 2018, 90, 10688–10694. 10.1021/acs.analchem.8b03201. 30139253 PMC6143422

[ref59] Tran Q. H.; Hoang D. H.; Song M.; Choe W.; Kang I.; Kim S. S.; Ha J. Melatonin and Doxorubicin Synergistically Enhance Apoptosis via Autophagy-Dependent Reduction of AMPKα1 Transcription in Human Breast Cancer Cells. Exp. Mol. Med. 2021, 53, 1413–1422. 10.1038/s12276-021-00675-y. 34584194 PMC8492618

[ref60] Hurvitz S. A.; McAndrew N. P.; Bardia A.; Press M. F.; Pegram M.; Crown J. P.; Fasching P. A.; Ejlertsen B.; Yang E. H.; Glaspy J. A.; Slamon D. J. A Careful Reassessment of Anthracycline Use in Curable Breast Cancer. npj Breast Cancer 2021, 7, 134 10.1038/s41523-021-00342-5. 34625570 PMC8501074

