# Supplementary material for: Hybrid Fluorescent Mass-Tag Nanotrackers as Universal Reagents for Long-Term Live-Cell Barcoding
Source: Anal Chem. 2022 Jul 22;94(30):10626–35. doi: 10.1021/acs.analchem.2c00795 (PMC9352147; doi:10.1021/acs.analchem.2c00795)
Supplement: Supplementary file 1 — ac2c00795_si_001.pdf [file ac2c00795_si_001.pdf]

SUPPORTING INFORMATION

HYBRID FLUORESCENT MASS-TAG NANOTRACKERS AS UNIVERSAL REAGENTS FOR LONG-TERM LIVE-CELL BARCODING

Antonio Delgado-Gonzalez[‡,a,b,c,d], Jose Antonio Laz-Ruiz[‡,a,b,c], Maria Victoria Cano-Cortés[a,b,c], Ying-Wen Huang[d], Veronica D. Gonzalez[d,†], Juan Jose Diaz-Mochon[a,b,c], Wendy J. Fantl[d,e,f,*], and Rosario M. Sanchez-Martin[a,b,c,*]

[a] *GENYO, Centre for Genomics and Oncological Research, Pfizer/University of Granada/Andalusian Regional Government, PTS Granada, Avda. Ilustración 114, 18016 Granada, Spain*

[b] *Department of Medicinal & Organic Chemistry and Excellence Research Unit of "Chemistry applied to Biomedicine and the Environment", Faculty of Pharmacy, University of Granada, Campus de Cartuja s/n, 18071 Granada, Spain*

[c] *Biosanitary Research Institute of Granada (ibs.GRANADA), University Hospitals of Granada-University of Granada, 18016 Granada, Spain*

[d] *Department of Urology, Stanford University School of Medicine, Stanford, CA 94305, USA*

[e] *Stanford Cancer Institute, Stanford University School of Medicine, Stanford, CA 94305, USA*

[f] *Department of Obstetrics and Gynecology, Stanford University School of Medicine, Stanford, CA 94304, USA*

[†] *Present address: 10X Genomics, Pleasanton, CA 94588, USA*

[‡] *These authors contributed equally to this work*

[*] Email: wjfantl@stanford.edu; rmsanchez@go.ugr.es

TABLE OF CONTENTS

1. **Supplementary Figures and Tables** S2
2. **General experimental methods** S5
   2.1. **Materials** S6
   2.2. **Equipment** S6
   2.3. **Synthesis of cross-linked polystyrene aminomethyl NPs (Naked-NPs (1))** S6
   2.4. **Characterization of NK-NPs (1)** S7
      2.4.1. **Solid content (SC) of the emulsion (%)** S7
      2.4.2. **Calculation of number of particles per gram** S7
      2.4.3. **Calculation of loading of NPs using Fmoc NPs test** S7
      2.4.4. **Qualitative ninhydrin test** S7
   2.5. **Determination of NPs concentration (NPs/µL) by spectrophotometric method** S8
   2.6. **General protocol for Fmoc deprotection** S8
3. **Synthesis of nanotrackers** S8
   3.1. **Synthesis of Cy5/Cy3-NTs (3)** S8
   3.2. **Synthesis of ^{106}Pd(II)-Cy5-NTs (4A) and ^{110}Pd(II)-Cy3-NTs (4B)** S9
   3.3. **Synthesis of ^{106}Pd(0)-Cy5-NTs (BC-1) and ^{110}Pd(0)-Cy3-NTs (BC-2)** S9
4. **Cell culture** S9
5. **Monitoring cellular internalization of nanotrackers by mass cytometry** S9
6. **Monitoring cellular internalization of nanotrackers by flow cytometry** S9
7. **Cellular uptake of hybrid-tag nanotrackers by confocal microscopy** S9
8. **Cell viability** S10
9. **Antibodies for mass cytometry** S10
10. **References** S10

1. Supplementary Figures and Tables

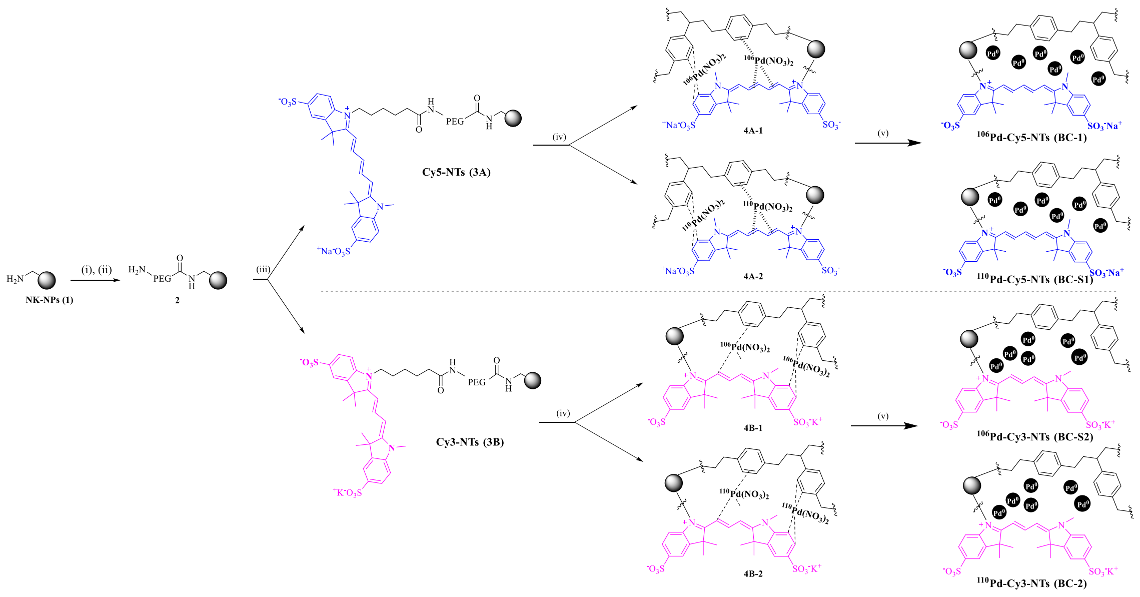

Scheme S1. General procedure for the functionalization of amino-loading polymeric nanoparticles to obtain hybrid-tag nanotrackers. Reagents and conditions: (i) Fmoc-NH-PEG-OH (15 eq), Oxyma (15 eq), DIC (15 eq), 2 h, 60 °C; (ii) 20% piperidine/DMF, 3 x 20 min, RT; (iii) sulfo-Cy5-NHS or sulfo-Cy3-NHS (0.005 µg/mL), DIPEA (1 eq), 16 h, RT; (iv) metal ($Pd(NO_3)_2$) isotopically pure (106 or 110) (1 mM) coordination via π-π stacking interaction, 15 h, RT; (v) Pd reduction, hydrazine/MeOH (1:5), 40 min, RT.

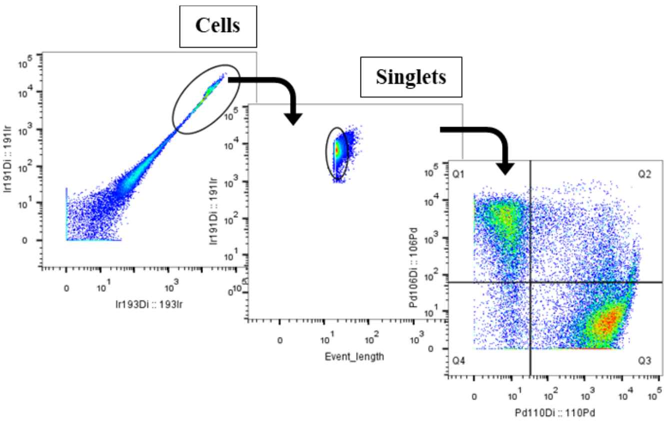

Figure S1. Gating strategy for mass cytometry analysis for identification of singlets. Firstly, cells are identified by DNA1 vs DNA2 (Ir191+/Ir193+) population; then, singlets are selected (Ir191 vs Event_length).

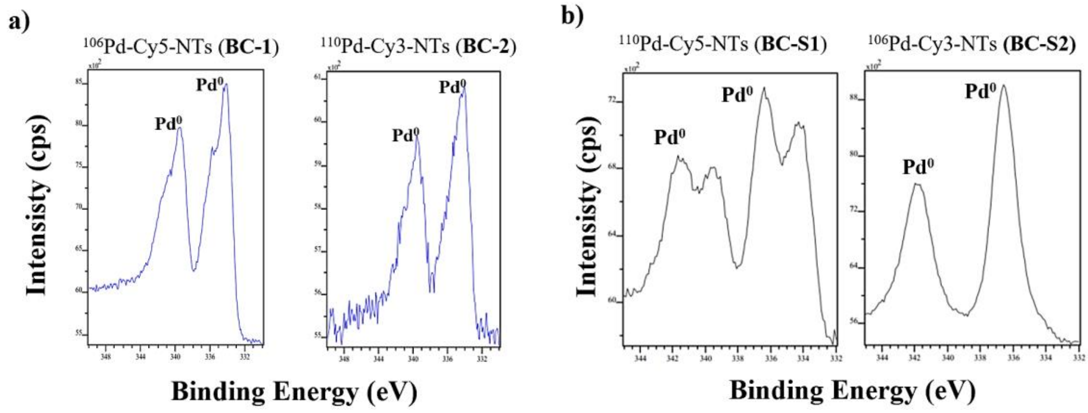

Figure S2. XPS spectra of **(a) BC-1** and **BC-2**, and **(b) BC-S1** and **BC-S2** hybrid-tag nanotrackers.

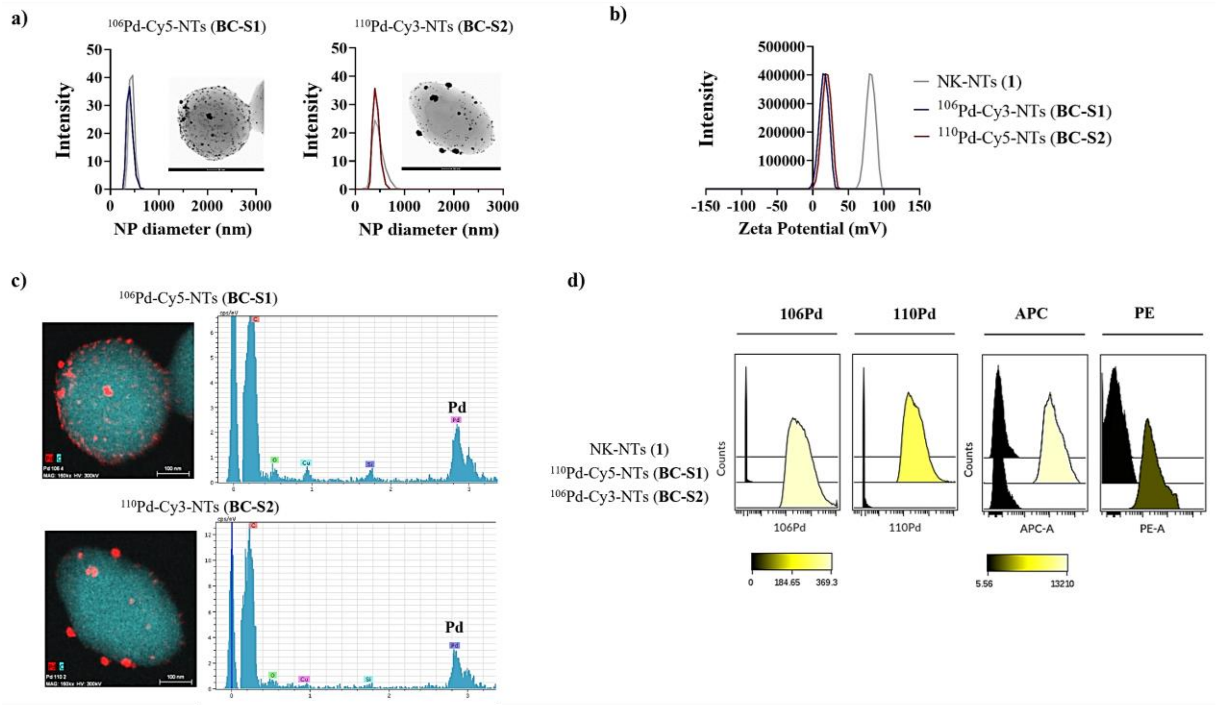

Figure S3. Physicochemical characterization of ^{110}Pd-Cy5-NTs (**BC-S1**) and ^{106}Pd-Cy3-NTs (**BC-S2**): **(a)** hydrodynamic diameter values of DLS and TEM image; **(b)** zeta potential values; **(c)** EDX analysis of Pd signal of the developed nanotrackers (carbon signal in blue, palladium signal in red); **(d)** histograms of Pd isotopes (left) and fluorophores signal (right) of the nanotrackers by mass and flow cytometry (NK-NPs (**1**) are non-conjugated nanoparticles control).

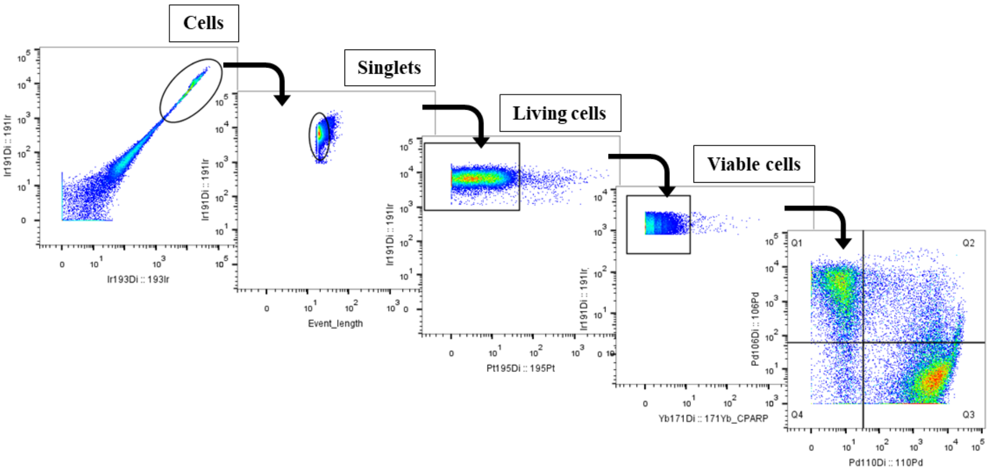

Figure S4. Gating strategy for mass cytometry analysis of viable cells. Firstly, cells are identified by DNA1 vs DNA2 (Ir191+/Ir193+) population; then, singlets are selected (Ir191 vs Event_length); after that, live cells (Ir191+/Pt195-), and finally non-apoptotic (viable) cells (Pt195-/cPARP(Yb171-)).

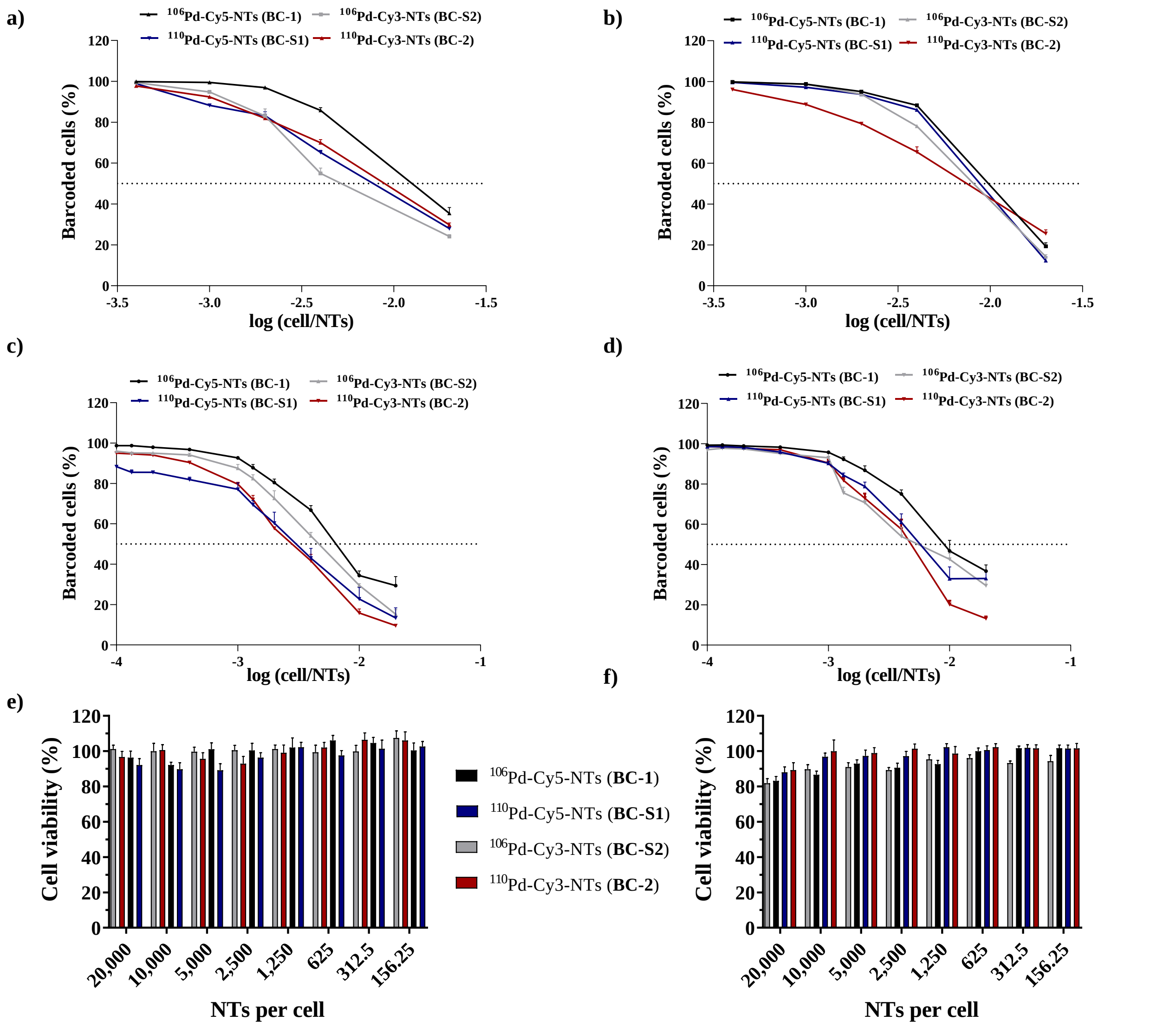

Figure S5. Internalization and viability assays of developed hybrid-tag nanotrackers. MDA-MB-231 and MCF-7 cell lines were selected. **(a)** Cellular uptake of MDA-MB-231 cells analyzed by CyTOF; **(b)** cellular uptake of MCF-7 cells analyzed by CyTOF; **(c)** cellular uptake of MDA-MB-231 cells analyzed by FACS; **(d)** cellular uptake of MCF-7 cells analyzed by FACS; Cell viability after 72 h of incubation with hybrid-tag nanotrackers measured by resazurin assay of **(e)** MDA-MB-231 cells and **(f)** MCF-7 cells.

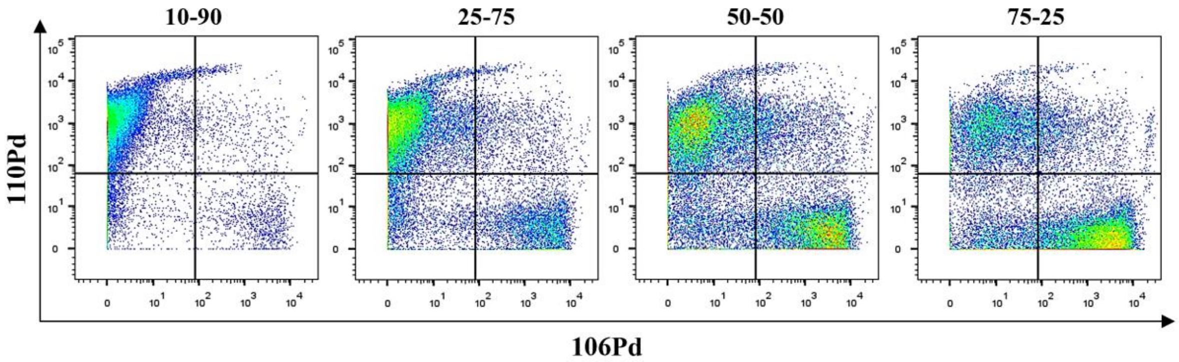

Figure S6. Cell cocultures of barcoded MDA-MB-231 and MCF-7 with **BC-1** and **BC-2** respectively by mass cytometry.

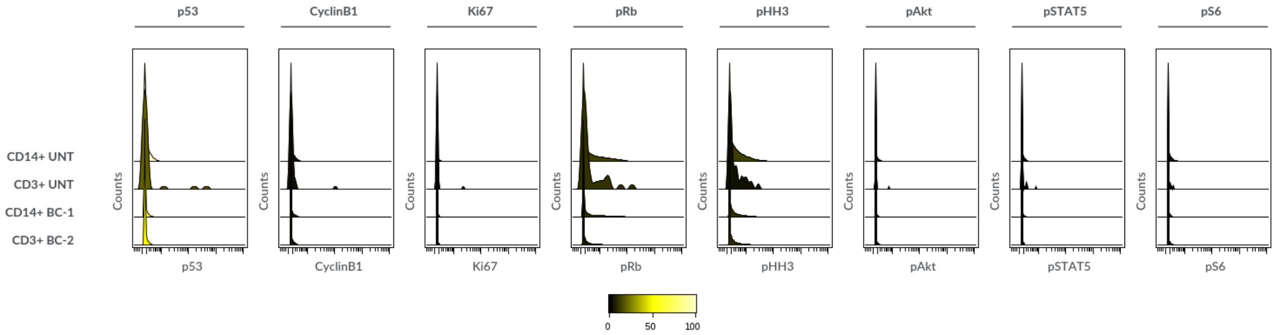

Figure S7. Mass cytometry analysis to assess cell damage of barcoded cells (CD14+BC-1 and CD3+BC-2). Monocytes (CD14+) and T cells (CD3+) without barcoding were used as control.

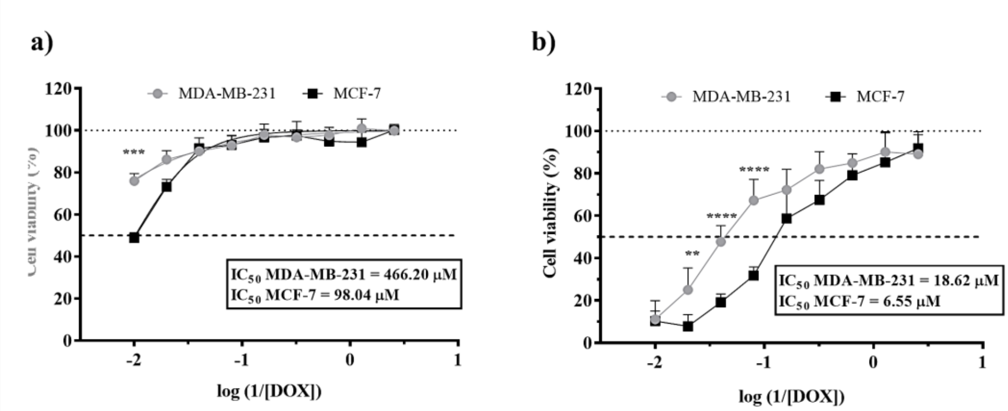

Figure S8. IC_{50} of MDA-MB-231 (gray) and MCF-7 (black) after **(a)** 6 h and **(b)** 24 h of incubation with doxorubicin.

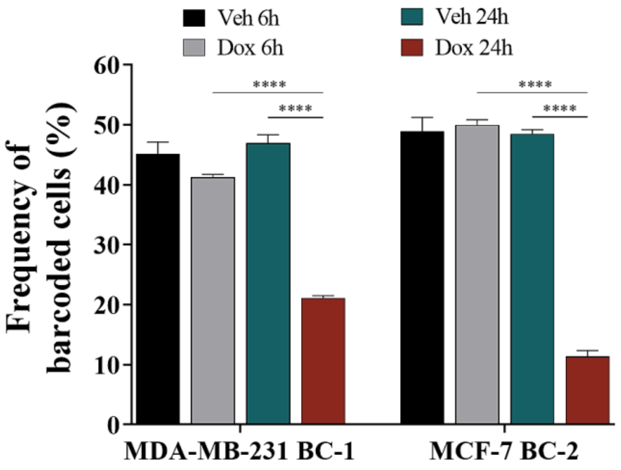

Figure S9. Frequencies of barcoded cells in coculture recovered after doxorubicin exposure compared to untreated (Veh) cells.

Table S1. Cells containing hybrid-tag nanotrackers after 3, 7, 10 and 14 days detected by **(a)** mass cytometry and **(b)** flow cytometry

a)

	% CELLS CONTAINING NTs	
**DAYS**	**MDA-MB-231 (BC-1)**	**MCF-7 (BC-2)**
**0**	95.70	98.03
**3**	81.83	97.43
**7**	55.00	79.47
**10**	33.40	63.93
**14**	16.33	34.77

b)

	% CELLS CONTAINING NTs	
**DAYS**	**MDA-MB-231 (BC-1)**	**MCF-7 (BC-2)**
**0**	96.37	96.03
**3**	83.20	93.77
**7**	58.33	80.80
**10**	38.07	63.93
**14**	19.67	38.10

Table S2. Mass cytometry antibody panel used in this study.

ISOTOPE	ANTIGENS	CLONE	PHOSPHORYLATION SITE	VENDOR
**In 115**	Vimentin	D21H3		CST
**Nd 146**	pATM	10H11.E12	pS1981	Milipore
**Sm 147**	pH2AX	JBW301	pS139	Milipore
**Nd 148**	CyclinB1	GNS-1		BD
**Sm 149**	pNFkB	K10-895.12.50	pS529	BD
**Nd 150**	pBcl2	5H2	pS70	CST
**Tb 159**	pAkt	D9E	pS473	CST
**Gd 160**	Sox2	O30-678		BD
**Dy 161**	c-Myc	D84C12		CST
**Dy 162**	pSTAT5	47/Stat5	pY694	BD
**Ho 165**	pRb	J112-906	pS807/pS811	BD
**Yb 171**	cPARP	F21-852	cleaved N214	BD
**Yb 172**	pS6	N7-548	pS235/pS236	BD
**Lu 175**	Total p53	1C12		CST
**Yb 176**	pHH3	HTA28	pS28	Biolegend

2. General experimental methods

2.1. Materials.

All solvents, chemicals and monoclonal antibodies were purchased from Sigma-Aldrich. Antibodies used for mass cytometry were purchased from different vendors (CST, Millipore, BD and BioLegend) listed in Table S1. Dulbecco's modified Eagle's medium (DMEM), l-glutamine, 1% penicillin/streptomycin, trypsin-EDTA, Dulbecco's Phosphate Buffered Saline (DPBS), and fetal bovine serum (FBS) were purchased from Gibco (Thermo Fisher Scientific). Cell Staining Medium was prepared in-house (DPBS with 0.5% BSA and 0.02% sodium azide).

2.2. Equipment

All mass cytometry experiments were performed using a Helios updated CyTOF2 (DVS Sciences, Fluidigm Co., CA, USA) except for titrations of metal-labeled antibodies which were performed in a CyTOF2 (DVS Sciences, Fluidigm Co., CA, USA). Flow cytometry experiments were performed on a FACSCanto II system (Becton Dickinson & Co., NJ, USA). The analysis of the results obtained in both cytometers was done using the Flowjo® 10 software and Cytobank. Cell viability was assayed using an M200 Nanoquant microplate reader to measure absorbance. Confocal microscopy images were obtained using a Zeiss LSM 710 confocal laser scanning microscope and Zeiss ZEN 2010 software for image acquisition. Transmission electron microscopy experiments were carried out using an ultra-high resolution FEI Titan G2 microscope with a XFEG Field Emission Gun operating at 300 kV. XPS spectra were obtained using a Kratos Axis Ultra-DLD X-ray photoelectron spectrometer equipped with an Al monochromatic X-ray source, over powdered nanoparticle samples.

2.3. Synthesis of cross-linked polystyrene aminomethyl NPs (Naked-NPs (1))

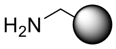

1

PVP (MW 29,000; 0.05 g; 1.7 µmol, Sigma-Aldrich) was dissolved in 92% ethanol/8% water for a final volume of 10 mL and deoxygenated via argon bubbling. AIBN (7 mg, 42.4 µmol) was dissolved in styrene (freshly washed, 0.5 mL) with VBAH (7 mg, 41.3 µmol) and DVB (freshly washed, 4.65 µL)[1]. The dispersion was deoxygenated with argon bubbling before the addition to the PVP/ethanol solution.

The mixture was stirred under argon for 1 h before heating to 68 °C for 15 h. Nanoparticles (NPs) were obtained by centrifugation (11,000 x g, 15 min) and washed with methanol (2 x 10 mL) and water (2 x 10 mL). Finally, NPs were stored in water (10 mL) at 4 °C[1].

Particle size distribution: mean diameter: 410.2 nm, PDI: 0.06

Loading (Ninhydrin test): 0.0368 mmol/g

Number of particles per gram: 2.77 x 10^{13}

2.4. Characterization of NK-NPs (1)

2.4.1. Solid content (SC) of the emulsion (%)

A known mass of a suspension of polystyrene NPs (0.5-1mg, suspended in water) was placed in a watch glass, covered with aluminum foil, dried at 25 °C for 15 h, weighed and reweighed to give the mass of NPs. The solid content was then calculated according to the following equation:

$$\% \, SC = \frac{M}{Vs} \, x \, 100$$

Equation S1. Solid content of NK-NPs (**1**).

Where m = mass of NPs (mg), Vs = Volume of suspension (µL).

CS: 3%, 3 mg of NPs in 100 µL of solution.

2.4.2. Calculation of number of particles per gram

$$N = {}^{6x10^{12}}/_{\pi x \rho x d^3}$$

Equation S2. Number of NK-NPs (**1**) per gram.

Where N: Number of particles/g for dry powder, ρ: Density of solid spheres (g/cm^3), which is 1 g/cm^3 for polystyrene, d: Mean diameter (nm).

Result: N= 2.77 x 10^{13} NTs per gram

2.4.3. Calculation of loading of NPs using Fmoc NPs test

Fmoc-(x)-NPs (where x is Fmoc-PEG-OH or Fmoc-Lys(Dde)-OH, etc) were resuspended in 1 mL of 20% piperidine in DMF (3 x 20 min) after which the beads were washed three times by centrifugation, and the supernatants combined. Loading was calculated according to the following equation:

$$Loading \left({}^{mmol}/_{g}\right) = \frac{(A_{302} x V)}{(\varepsilon_{302} x d x W)} x 1000$$

Equation S3. Amino-loading of nanoparticles.

Where A_{302}: Absorbance measured at 302 nm, V: Volume of combined supernatants (mL), ε_{302}: Molar Extinction Coefficient (7800 $M^{-1}cm^{-1}$) and W: Mass of beads (mg).

Result: Loading (Fmoc test): 0.0368 mmol/g

2.4.4. Qualitative ninhydrin test

Reaction completion control was determined by qualitative ninhydrin test (Kaiser test)[2]. Small samples of NPs (12 µL, 3% SC) were transferred to 0.5 mL capacity eppendorf and washed once with methanol and centrifuged after which 6 µL of reagent A and 2 µL of reagent B were added. The suspension was mixed well and heated to 100 ºC for 3 min. Blue stained resin beads indicate the presence of primary amines.

2.5. Determination of NPs concentration (NPs/µL) by spectrophotometric method

NPs concentration (NPs per microliter) was determined by a spectrophotometric method as described previously[3]. Briefly, measurement of turbidity optical density at 600 nm of polystyrene NP suspensions was performed, based on nephelometric principles. Light going through NP suspensions is scattered via reflection, refraction and diffraction phenomena and the intensity of the scattered light, which is proportional to number of NPs in suspension, is recorded by standard spectrophotometers. In this way, calibrate standard curves were obtained for amino-methyl cross-linked polystyrene NPs of 410 nm by NP known concentrations. Calibration curves fitted linear regression models by which the number of NPs per microliter corresponding to one unit of OD600 for each size could be determined. Thus, these curves using initial batches of NP suspensions permitted us to estimate the number of NPs in final batches, which underwent multiple handling procedures, by OD600 measurement of 1 µL (**Figure S10**).

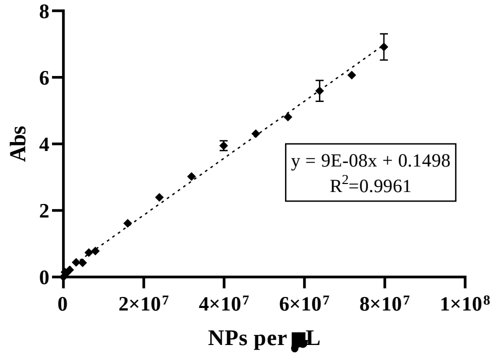

Figure S10. Calibration standard curve of concentration of nanoparticles (OD 600).

2.6. General protocol for Fmoc deprotection

Fmoc deprotection was achieved by treating NPs with 20% piperidine/DMF (1 mL; 3 x 20 min). NPs were obtained by centrifugation and subsequent wash with DMF (3 x 1 mL), MeOH (3 x 1 mL), and deionised water (3 x 1 mL).

3. Synthesis of nanotrackers

3.1. Synthesis of Cy5/Cy3-NTs (3)

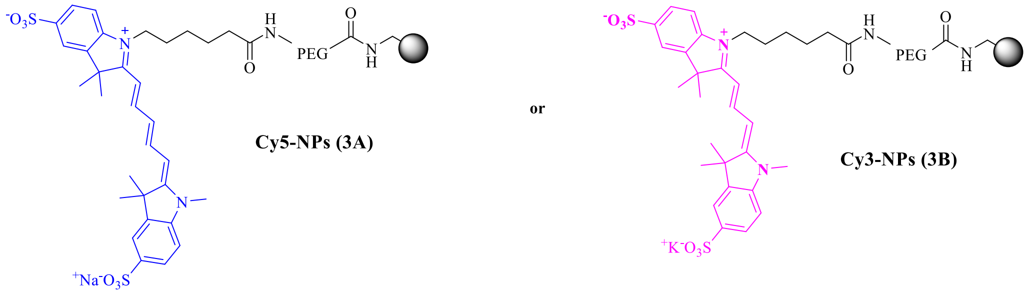

NK-NPs (**1**) nanoparticles were first conditioned by washing them three times with 1 mL of DMF each time through suspension-centrifugation cycles (13,400 rpm, 3 min). Next, to conjugate the PEG spacer to NTs, Fmoc–PEG–OH (15 eq) was dissolved in DMF (1 mL) with oxyma (15 eq) and DIC (15 eq). The mixture was stirred at RT for 10 min. Then, the solution was added to dry NTs, and the suspension was left to stir at 1,400 rpm at 60 °C for 2 h. Subsequently, NTs were washed with three successive suspension-centrifugation cycles (13,400 rpm, 3 min) to obtain Fmoc-PEGylated NTs. Fmoc was removed using a 20% piperidine/DMF solution (3 × 20 min) and sequential washing steps, as described above. Next, 50 µL of a sulfo-Cy5/Cy3-NHS ester solution (1 eq) was mixed separately with DIPEA (1 eq) before being added to dry PEGylated NTs, which were then suspended. The suspension was stirred at 1,000 rpm at RT for 14 h in the dark. Afterwards, the NTs were washed with three cycles of suspension-centrifugation with DMF (13,400 rpm, 3 min), then other three cycles in MeOH, and finally resuspended in mQ H_2O to obtain Cy5-NTs (**3A**) and Cy3-NTs (**3B**).

3.2. Synthesis of ^{106}Pd(II)-Cy5-NTs (4A) and ^{110}Pd(II)-Cy3-NTs (4B)

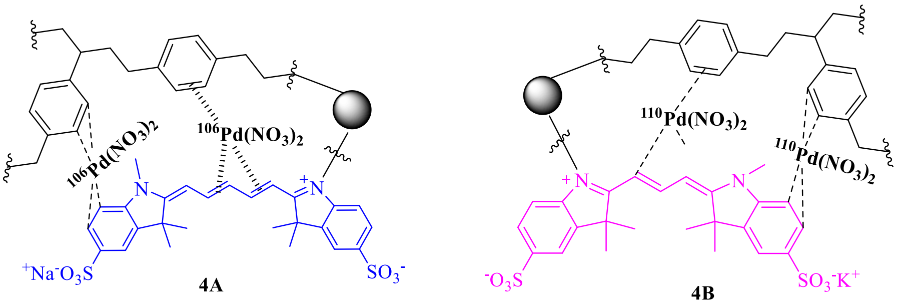

Next, 100 µL of a 10 mM Pd(NO$_3$)$_2$ (isotopically pure (^{106}Pd or ^{110}Pd) in mQ H_2O was added to dry NTs, and they were stirred at 1,000 rpm at RT for 14 h in the dark. Upon reaction completion, the NTs were washed with three successive suspension-centrifugation cycles with water, before finally being suspended in water (50 µL) to obtain ^{106}Pd-Cy5-NTs (**4A**) and ^{110}Pd-Cy3-NTs (**4B**).

Following the same protocol, ^{110}Pd-Cy5-NTs (**S4A**), ^{106}Pd-Cy3-NTs (**S4B**) were obtained.

3.3. Synthesis of ^{106}Pd(0)-Cy5-NTs (BC-1) and ^{110}Pd(0)-Cy3-NTs (BC-2)

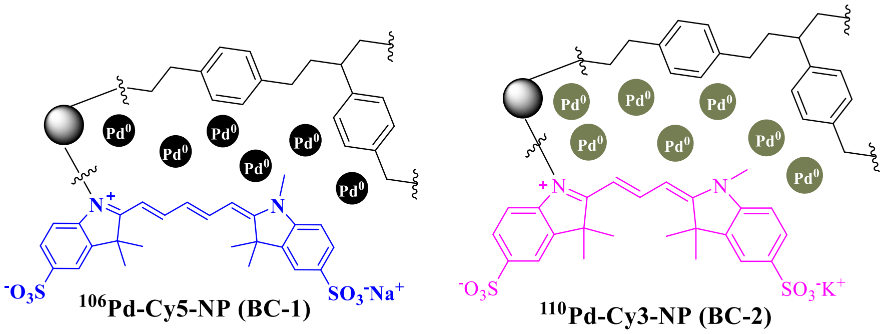

Pd(II) was reduced to Pd(0) by adding 150 µL of 10% hydrazine in methanol. The reaction mixture was left to stir at RT for 30 min. NTs were then washed with three suspension-centrifugation cycles with methanol (13,400 rpm, 5 min) and water (13,400 rpm, 8 min), before finally being suspended in water[4], obtaining ^{106}Pd-Cy5-NTs (**BC-1**) and ^{110}Pd-Cy3-NTs (**BC-2**).

Same protocol was applied to obtain ^{110}Pd-Cy5-NTs (**BC-S1**) and ^{106}Pd-Cy3-NTs (**BC-S2**).

4. Cell culture.

The cell bank of the Centre for Scientific Instrumentation of the University of Granada (CIC-UGR) provided cell lines. MDA-MB-231 and MCF-7 cell lines (breast cancer) were cultured in DMEM, supplemented with 10% (vol/vol), FBS, 1% l-glutamine and 1% penicillin/streptomycin. Cells were maintained at 37 °C, 5% CO_2, and 95% relative humidity, and were regularly tested negative for mycoplasma infection.

5. Monitoring cellular internalization of nanotrackers by mass cytometry.

Breast cancer cell lines (MDA-MB-231 and MCF-7) were incubated with hybrid-tag nanotrackers ranging in concentration from 50 – 2,500 NTs/cell. After 3 hours, cells were washed with serum-free media. Then, they were incubated with cisplatin solution (Sigma®) (25 µM final concentration) in serum-free media for 1 min after which cells were quenched with serum-containing media[18]. Cells were washed with DPBS, detached with trypsin, fixed in 1.6% paraformaldehyde (PFA) at room temperature (RT) for 10 min and incubated with 125 nM Cell-ID™ Intercalator-Ir (Fluidigm) solution at 4°C for 15 h. Cells were washed with CSM, followed by Maxpar® water. They were suspended in 0.1x EQ bead solution (Fluidigm) prior to introduction into the mass cytometer (Helios, Fluidigm). The gating strategy used for the analysis of viable cells is specified in **Figure S1**.

6. Monitoring cellular internalization of nanotrackers by flow cytometry.

MDA-MB-231 and MCF-7 cells were incubated with hybrid-tag nanotrackers at different concentrations, ranging from 50 to 10,000 NTs/cell. After 3 h, cells were washed with DPBS and separated with Trypsin at 37 °C for 5 min. Then, each sample was fixed in PFA at a final concentration of 2% at RT for 10 min. Samples were analyzed by flow cytometry. FCS datasets were analyzed using Cytobank Community software.

7. Cellular uptake of hybrid-tag nanotrackers by confocal microscopy

Cells were seeded on coverslips and incubated with 2,500 NTs per cell for 3 h. After incubation, media was removed, and cells were washed with DPBS and fixed in 4% PFA at RT for 10 min, protected from light. Samples were analyzed by confocal microscopy.

8. Cell viability

The cellular viability was evaluated using the resazurin assay protocol (Sigma®). Cells were seeded in 96-well plates and incubated with a range of hybrid-tag nanotrackers up to 20,000 NTs/cell for 72 h. After the addition of resazurin, the plate was directly measured at 570 nm. Viability was normalized respect to untreated cells (100%).

9. Antibodies for mass cytometry.

Antibodies (**Table S2**) in carrier-free PBS were conjugated to metal-chelated polymers (MaxPAR antibody conjugation kit, Fluidigm) according to the manufacturer's protocol. Metal-labeled antibodies were diluted to 0.2–0.4 mg/mL in antibody stabilization solution (CANDOR Biosciences) and stored at 4 °C. Each antibody was titrated using cell lines and primary human samples as positive and negative controls[5]. Antibody concentrations in experiments were based on an optimal signal-to-noise ratio.

10. References

¡Error! Referencia de hipervínculo no válida.(1) Unciti-Broceta, A.; Johansson, E. M. V.; Yusop, R. M.; Sánchez-Martín, R. M.; Bradley, M. Synthesis of Polystyrene Microspheres and Functionalization with Pd0 Nanoparticles to Perform Bioorthogonal Organometallic Chemistry in Living Cells. *Nat. Protoc.* **2012**, *7* (6), 1207–1218.

(2) Wellings, D. A.; Atherton, E. Standard Fmoc Protocols. *Methods Enzymol.* **1997**, *289*, 44–67.

(3) Unciti-Broceta, J. D.; Cano-Cortés, V.; Altea-Manzano, P.; Pernagallo, S.; Díaz-Mochón, J. J.; Sánchez-Martín, R. M. Number of Nanoparticles per Cell through a Spectrophotometric Method - A Key Parameter to Assess Nanoparticle-Based Cellular Assays. *Sci. Rep.* **2015**, *5*.

(4) Delgado-Gonzalez, A.; Garcia-Fernandez, E.; Valero, T.; Victoria Cano-Cortes, M.; Ruedas-Rama, M. J.; Unciti-Broceta, A.; Sanchez-Martin, R. M.; Diaz-Mochon, J. J.; Orte, A. Metallofluorescent Nanoparticles for Multimodal Applications. *ACS Omega* **2018**, *3* (1), 144–153.

(5) Gonzalez, V. D.; Samusik, N.; Chen, T. J.; Savig, E. S.; Aghaeepour, N.; Quigley, D. A.; Huang, Y. W.; Giangarrà, V.; Borowsky, A. D.; Hubbard, N. E.; Chen, S. Y.; Han, G.; Ashworth, A.; Kipps, T. J.; Berek, J. S.; Nolan, G. P.; Fantl, W. J. Commonly Occurring Cell Subsets in High-Grade Serous Ovarian Tumors Identified by Single-Cell Mass Cytometry. *Cell Rep.* **2018**, *22* (7), 1875–1888.
